# Immunomodulatory Functions of Adipose Mesenchymal Stromal/Stem Cell Derived From Donors With Type 2 Diabetes and Obesity on CD4 T Cells

**DOI:** 10.1093/stmcls/sxad021

**Published:** 2023-03-22

**Authors:** Marwa Mahmoud, Miia Juntunen, Amna Adnan, Laura Kummola, Ilkka S Junttila, Minna Kelloniemi, Tuula Tyrväinen, Heini Huhtala, Abeer I Abd El Fattah, Khalda Amr, Alaa Mohamad El erian, Mimmi Patrikoski, Susanna Miettinen

**Affiliations:** Adult Stem Cell Group, Faculty of Medicine and Health Technology, Tampere University, Tampere, Finland; Stem Cell Research Group, Medical Research Centre of Excellence, National Research Centre, Cairo, Egypt; Department of Medical Molecular Genetics, Human Genetics and Genome Research Division, National Research Centre, Cairo, Egypt; Adult Stem Cell Group, Faculty of Medicine and Health Technology, Tampere University, Tampere, Finland; Research, Development, and Innovation Centre, Tampere University Hospital, Tampere, Finland; Adult Stem Cell Group, Faculty of Medicine and Health Technology, Tampere University, Tampere, Finland; Research, Development, and Innovation Centre, Tampere University Hospital, Tampere, Finland; Biodiversity Interventions for Well-being, Faculty of Medicine and Health Technology, Tampere University, Tampere, Finland; Cytokine Biology Research Group, Faculty of Medicine and Health Technology, Tampere University, Tampere, Finland; Northern Finland Laboratory Centre (NordLab), Oulu, Finland; Research Unit of Biomedicine, University of Oulu, Oulu, Finland; Fimlab Laboratories, Tampere, Finland; Department of Plastic and Reconstructive Surgery, Tampere University Hospital, Tampere, Finland; Department of Gastroenterology and Alimentary Tract Surgery, Tampere University Hospital, Tampere, Finland; Faculty of Social Sciences, University of Tampere, Tampere, Finland; Department of Biochemistry, Faculty of Pharmacy (Girls), Al-Azhar University, Cairo, Egypt; Stem Cell Research Group, Medical Research Centre of Excellence, National Research Centre, Cairo, Egypt; Department of Endocrine Surgery, National Institute of Diabetes and Endocrinology, Cairo, Egypt; Research, Development, and Innovation Centre, Tampere University Hospital, Tampere, Finland; Obesity Research Unit, Research Program for Clinical and Molecular Metabolism, Faculty of Medicine, University of Helsinki, Finland; Finnish Red Cross Blood Service, Advanced Cell Therapy Centre, Helsinki, Finland; Adult Stem Cell Group, Faculty of Medicine and Health Technology, Tampere University, Tampere, Finland; Research, Development, and Innovation Centre, Tampere University Hospital, Tampere, Finland

**Keywords:** adipose mesenchymal stromal/stem cell, immunomodulatory, type 2 diabetes, obesity, cytokines, CD4 T cell

## Abstract

For adipose stromal/stem cell (ASCs)-based immunomodulatory therapies, it is important to study how donor characteristics, such as obesity and type 2 diabetes (T2D), influence ASCs efficacy. Here, ASCs were obtained from 2 groups, donors with T2D and obesity (dASCs) or nondiabetic donors with normal-weight (ndASCs), and then cultured with anti-CD3/CD28-stimulated allogeneic CD4 T cells. ASCs were studied for the expression of the immunomodulators CD54, CD274, and indoleamine 2, 3 dioxygenase 1 (IDO) in inflammatory conditions. CD4 T cells cultured alone or in cocultures were assessed to evaluate proliferation, activation marker surface expression, apoptosis, the regulatory T cells (Tregs; CD4^+^ CD25^high^ FOXP3^+^) frequency, and intracellular cytokine expression using flow cytometry. Modulation of T-cell subset cytokines was explored via ELISA. In inflammatory conditions, the expression of CD54, CD274, and IDO was significantly upregulated in ASCs, with no significant differences between ndASCs and dASCs. dASCs retained the potential to significantly suppress CD4 T-cell proliferation, with a slightly weaker inhibitory effect than ndASCs, which was associated with significantly reduced abilities to decrease IL-2 production and increase IL-8 levels in cocultures. Such attenuated potentials were significantly correlated with increasing body mass index. dASCs and ndASCs comparably reduced CD4 T-cell viability, HLA-DR expression, and interferon-gamma production and conversely increased CD69 expression, the Tregs percentage, and IL-17A production. Considerable amounts of the immunomodulators prostaglandin E2 (PGE2) and IL-6 were detected in the conditioned medium of cocultures. These findings suggest that ASCs obtained from donors with T2D and obesity are receptive to the inflammatory environment and able to modulate CD4 T cells accordingly.

## Graphical Abstract

**Graphical Abstract:**
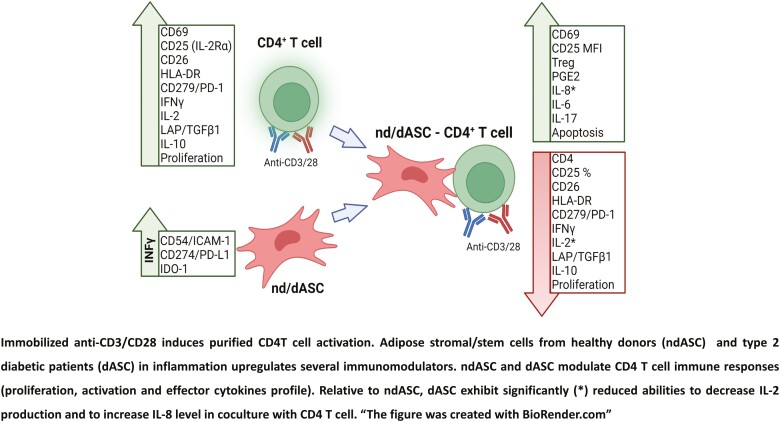

**Significance Statement**

The therapeutic utilization of adipose stromal/stem cells (ASCs) to treat inflammatory diseases relies mainly on the immunoregulatory activities of these cells. An environment characterized by inflammation and oxidative stress may affect the immunomodulatory function of ASCs from donors with type 2 diabetes (T2D) and obesity (dASCs). In our study, dASCs retained the potential to modulate CD4 T cells and express immunomodulators in inflammatory conditions. However, increasing body mass index (BMI) slightly but significantly attenuated ASC immunosuppressive functions. Despite the immunosuppressive functions of dASCs in vitro, the possible utilization of dASCs in therapies requiring immunomodulation needs further investigation that considers donor characteristics such as BMI.

## Introduction

Type 2 diabetes (T2D) is a hyperglycemic state in which imbalanced metabolic and inflammatory pathways are integrated.^1^ This disease is often associated with obesity and develops when overnutrition and physical inactivity combined with genetic susceptibility to cause insulin resistance (IR).^2,3^ Obesity induces a chronic state of low-level systemic inflammation;^4,5^ in this context, the infiltration of T cells in adipose tissue (AT), in concert with macrophage accumulation, is a major player.^6^ AT is a plentiful, easily accessible source of mesenchymal stromal/stem cells (MSCs), with minimal risks for donors.^7^ Adipose MSCs, here referred to as adipose stromal/stem cells (ASCs), represent a promising agent for inflammatory disease treatment.^8,9^ The anti-inflammatory functions of ASCs have been documented in T2D models to regenerate β cells and ameliorate IR.^10,11^ Furthermore, ASCs possess antioxidative properties to mitigate oxidative stress, such as that associated with obesity and T2D.^12^

ASCs have a plastic immunomodulatory capacity to regulate T cells^13-18^ via direct^16,17^ and paracrine^19-23^ mechanisms. Among the key paracrine mediators are indoleamine 2, 3-dioxygenase 1 (IDO)^20-22^ and prostaglandin E2 (PGE2).^13,23^ The induction^24^ or expansion^25^ of Tregs has also been reported. An inflammatory stimulus, such as interferon-gamma (IFN-γ), or crosstalk with active immune cells is necessary for MSCs to exert their immunosuppressive effects.^14^ Despite the hypoimmunogenic nature of MSCs and the rise of allogeneic MSC therapy,^9,26^ autologous MSCs may be the relatively safe choice from an immunological point of view.^26,27^ The inflammation and oxidative stress associated with obesity and T2D may alter the immunophenotype, immunosuppressive functions, and antioxidative properties of ASCs.^4,28-30^

In the present study, ASCs were isolated from nondiabetic donors with normal weight (ndASCs) and donors with T2D and obesity (dASCs). Then, the characteristics of ndASCs and dASCs, focusing on their suppressive capacity against CD4 T-cell immune responses, were compared. The levels of reactive oxygen species (ROS) generated by ndASCs and dASCs under basal and stress conditions were also measured. Both ASC groups were studied for the expression of CD54/intracellular adhesion molecule (ICAM), CD274/programmed death ligand 1 (PD-L1), and IDO in the context of inflammation induced by IFN-γ or coculture with preactivated peripheral blood mononuclear cells (PBMCs). ndASCs or dASCs were cultured directly with anti-CD3/CD28-stimulated allogeneic CD4 T cells. Following monoculture or coculture, CD4 T cells were analyzed by flow cytometry to evaluate proliferation, activation, apoptosis, the Tregs frequency, and intracellular cytokine production. The cytokine profile of CD4 T helper (Th) cells, including Th1 cells, Th17 cells, and Tregs, was explored by ELISA. Delineating the basic characteristics and anti-inflammatory capability of dASCs in vitro is an essential preliminary step for preclinical and clinical research to unravel dASC suitability for use in therapies requiring immunomodulation.

## Subjects and Methods

### Subjects

Subcutaneous AT specimens were retrieved from 2 donor groups. One group included donors with T2D and obesity (*n* = 9, median BMI: 42.56, median age: 57] who underwent gastric bypass surgery at the Tampere University Hospital (TAUH), Department of Gastroenterology and Alimentary Tract Surgery, and another group included nondiabetic donors (*n* = 5, median BMI: 25.2, median age: 42) who underwent plastic surgery at the TAUH, Department of Plastic Surgery. The tissue samples were obtained with the donors’ written informed consent and processed under ethical approval by the Ethics Committee of the Expert Responsibility area of TAUH (R15161). Donor characteristics are shown in Supplementary Table S1. The nondiabetic donors and donors with T2D and obesity had median ages (42 and 57, respectively, *P* = .012) and median BMI (25.2 and 42.6, respectively, *P* = .001). Human blood samples, used for buffy coat isolation, were obtained from the Finnish Red Cross Blood Service, and the study was conducted in accordance with the Declaration of Helsinki 1975, revised in Hong Kong 1989.

### Cell Isolation and Culture

Human ASCs were isolated from the subcutaneous AT of nondiabetic and diabetic donors as previously described.^31^ ASCs were cultured in basal medium (BM) composed of *α*-modified minimum essential medium (α-MEM; Gibco, Life Technologies, Carlsbad, CA, USA), 5% human serum (HS type AB male, Serana, Brandenburg, Germany), 100 U/mL penicillin, and 100 µg/mL streptomycin (Pen/Strep; Lonza, Bio Wittaker, Verviers, Belgium). The cells were expanded to passages (P) 4-5.

To assess the effect of inflammation on ASC immunomodulatory activity, ASCs were primed with recombinant human IFN-γ (10 ng/mL) (R&D Systems, Minneapolis, MN, USA) for 48 h. Conditioned medium (CM) from IFN-γ-primed ASCs was collected and stored at −80 °C.

PBMCs were isolated by gradient centrifugation of heparinized blood obtained from healthy donors; the blood was diluted with Dulbecco’s phosphate-buffered saline (DPBS) (Lonza, USA) and overlaid on Ficoll Paque Plus (density: 1.077 g/mL; GE Healthcare, UK). CD4 T-cell lines (*n* = 12) were purified from freshly prepared PBMCs by magnetic activated cell sorting (MACS) using a human CD4 T-cell isolation kit (Miltenyi Biotec, Bergisch Gladbach, Germany). In addition, naïve CD4 T-cell (CD4^+^ CD25^−/dim^) lines (*n* = 4) were purified using a naïve CD4 T-cell isolation kit (Miltenyi Biotec) according to the manufacturer’s instructions. After isolation, the cells were cryopreserved in the gaseous phase of liquid nitrogen.

### Phenotypic Characterization of ASCs

To verify the phenotypic characteristics of ASCs according to the International Society for Cell & Gene Therapy (ISCT)^32^ and the International Federation for Adipose Therapeutics and Science (IFATS),^33^ ndASCs (*n* = 5) or dASCs (*n* = 9) at P5 were assessed for the expression of the surface markers CD13, CD19, CD29, CD31, CD34, CD36, CD44, CD45R0, CD73, CD90, CD105, CD235a, and HLA-DR using a CytoFlex S flow cytometer (Beckman Coulter). Additionally, we monitored the expression of CD54 and CD146 (Supplementary Table S2). The percentage of positive cells and median fluorescence intensity (MFI) of each molecule were determined. The MFI reflects the expression level of the marker for one cell.

### Surface Expression of Immune-Related Markers Under Inflammatory Conditions

The expression levels of the surface markers HLA-ABC, HLA-DR, CD86, CD40, CD54, and CD274 on ASCs under inflammatory conditions were also investigated. Two different inflammatory conditions for ASCs were applied: priming with IFN-γ (10 ng/mL) for 48 h and coculture with anti-CD3/CD28-prestimulated PBMCs (Section: ASC/CD4 T-cell coculture) for 72 h.

### Functional Verification of ASCs

To verify the mesenchymal origin of ASCs, ndASCs (*n* = 5) and dASCs (*n* = 9) were induced to differentiate into osteogenic, adipogenic, and chondrogenic lineages. The methodology for the differentiation assays is illustrated in Supplementary File 1.

### ASC Proliferation Analysis

ndASCs (*n* = 4) and dASCs (*n* = 9) at P 4/5 were seeded in 75-cm^2^ flasks (Nunc, Thermo Scientific, Carlsbad, CA, USA) at a density of 2000 cells/cm^2^, and the BM was changed every third day. On the seventh day, the cells were harvested and counted using a TC20 automated cell counter (Bio-Rad). The population doubling time (PDT) was calculated using the formula PDT = *T* × log2/(logNf − logNi), where *T* is the culture time, Ni is the initial cell number, and Nf is the final harvested cell number.^34^ Experiments were repeated twice. Proliferation was also assessed with a Cell Counting Kit-8 (CCK-8) assay (Dojindo Laboratories, Japan).^35^

### Detection of ROS Generation by ASCs

Basal and tert-butyl hydroperoxide (TBHP; Sigma Aldrich, St. Louis, MO, USA)-induced ROS generated by ASCs were assessed by labeling cells using—the DCFDA/H2DCFDA-Cellular ROS Assay Kit (Abcam, Cambridge, UK). To assess the antioxidative potential of ndASCs vs. that of dASCs, labeled ndASCs and dASCs were treated with the oxidative agent TBHP (110 µm) for 2 h, and then the fluorescence intensity was measured using a microplate reader (Victor 1429 Multilabel Counter, Wallac, Turku, Finland).

### IDO Protein Assessment

The expression of the IDO protein, in naïve and IFN-γ-primed ndASCs and dASCs, was quantified using the Human IDO SimpleStep ELISA Kit (Abcam) according to the manufacturer’s instructions. The protein content of cell extracts was measured using a Pierce BCA protein assay kit (Thermo Fisher Scientific). IDO concentrations were normalized to a 20-µg/mL protein extract.

### ASC/CD4 T-Cell Coculture for the Detection of Immunosuppressive Functions and Immunogenicity

To study immunosuppressive functions, ndASCs and dASCs at P5 (50-60 × 10^3^) were separately cultured in anti-human CD3 (1 µg/mL) and anti-human CD28 (2 µg/mL) (Miltenyi Biotec)-precoated 12-well plates (Corning CellBIND, Corning Inc., NY, USA) overnight. Purified CD4 T cells at a density of 8 × 10^5^-10^6^ cells/mL were then added to the adherent ASCs (1:10-1:20, ASC: CD4 T-cell ratio). In parallel, stimulated CD4 T-cell monocultures were conducted in anti-CD3/CD28 precoated plates as controls. Cells were cultured in RPMI 1640 medium (Gibco) supplemented with 5% HS for 5 days. In the CD4 T-cell proliferation assay, recombinant human IL-2 (Miltenyi Biotec) (50 IU/mL) was added to the culture medium. For ASC immunogenicity experiments, ndASCs and dASCs were separately seeded in noncoated 12-well plates and cocultured for 6 days with allogeneic naïve CD4 T cells (1:10, ASC:CD4 T-cell ratio).

### CD4 T-Cell Proliferation, Apoptosis, Cell Cycle, and Activation Marker Analyses

A CytoFlex S flow cytometer and FlowJo v.10.8.1 (Tree Star, Ashland, OR) were used to monitor and analyze the proliferation, apoptosis, cell cycle, and activation marker expression of stimulated CD4 T cells in monocultures and cocultures.

For the proliferation assay, CD4 T cells in monocultures and cocultures were labeled with CellTrace Violet (1-2 µm) (CTV; Invitrogen, Thermo Fisher Scientific) following the manufacturer’s instructions. On the fifth day of culture, the T cells were harvested, stained with fixable viability stain 780 (FVS780, BD Biosciences Horizon, San Diego, CA) and anti-CD4-FITC, and then analyzed.

To detect apoptosis, annexin V-APC/propidium iodide (PI) (Invitrogen) dual staining was performed following the manufacturer’s instructions. The detected percentages of Annexin V^+^/PI^−^, Annexin V^+^/PI^+^, and Annexin V^−^/PI^+^ cells represent the percentages of cells in early apoptosis, late apoptosis, and necrosis, respectively.

For the cell cycle analysis, a red cell cycle assay kit (Abcam) was used following the manufacturer’s instructions. To assess the CD4 T-cell activation status, cells were stained with FVS780 and human anti-CD4-FITC, in addition to 1 or 2 of the following antibodies: anti-CD69-PE (eBioscience), anti-CD25-APC, anti-HLA-DR-BV605 (BD Horizon), anti-CD26-PE, and anti-CD279-PerCP. All antibodies were from BD Biosciences Pharmingen unless another supplier is indicated.

### Tregs Detection and Intracellular Cytokine Analysis

To detect Tregs, intracellular staining of CD4^+^ CD25^+^ T cells was performed using a human FOXP3 buffer set (BD Biosciences) and anti-human FOXP3-PE antibody (BD Pharmingen) according to the manufacturer’s instructions. For intracellular cytokine analysis, CD4 T cells cultured alone or cocultured with ASCs were restimulated with leukocyte activation cocktail (BD Biosciences) for 2-3 h on the day of analysis. The T cells were then harvested, fixed, and permeabilized using a BD Cytofix/Cytoperm kit (BD Biosciences). The T cells were stained with BV605-conjugated anti-human IFN-gamma or APC-conjugated anti-human IL-10 (BD Biosciences) and analyzed using a CytoFlex S flow cytometer.

### Measurement of Cytokine Concentrations by ELISA

CD4 T cells in monocultures or cocultures with ndASCs or dASCs (ASC: CD4 T-cell ratio, 1:15) were seeded in anti-CD3/CD28-precoated 6-well plates (Costar, Corning) to assess the effect of ASCs on Th-cell polarization. The cell-free culture supernatants were collected on the fifth day and stored at −80 °C. Human IFN-gamma, IL-2, IL-17, IL-6, IL-8, PGE2, IL-10, and LAP (TGF-β1) concentrations were assessed with ELISA kits (Thermo Fisher Scientific) following the manufacturer’s instructions. Data from 2 different T-cell lines were considered.

### Statistical Analysis

Statistical analysis was performed using GraphPad Prism software version 9 (GraphPad Inc.). Data are presented as the median with the range. *P* values were calculated using the Mann-Whitney *U* test. Spearman and Pearson correlation coefficients were used to correlate between studied variables. *P* ≤ .05 was considered significant.

## Results

### Proliferation and ROS Levels of ndASCs and dASCs

**Figure 1.**
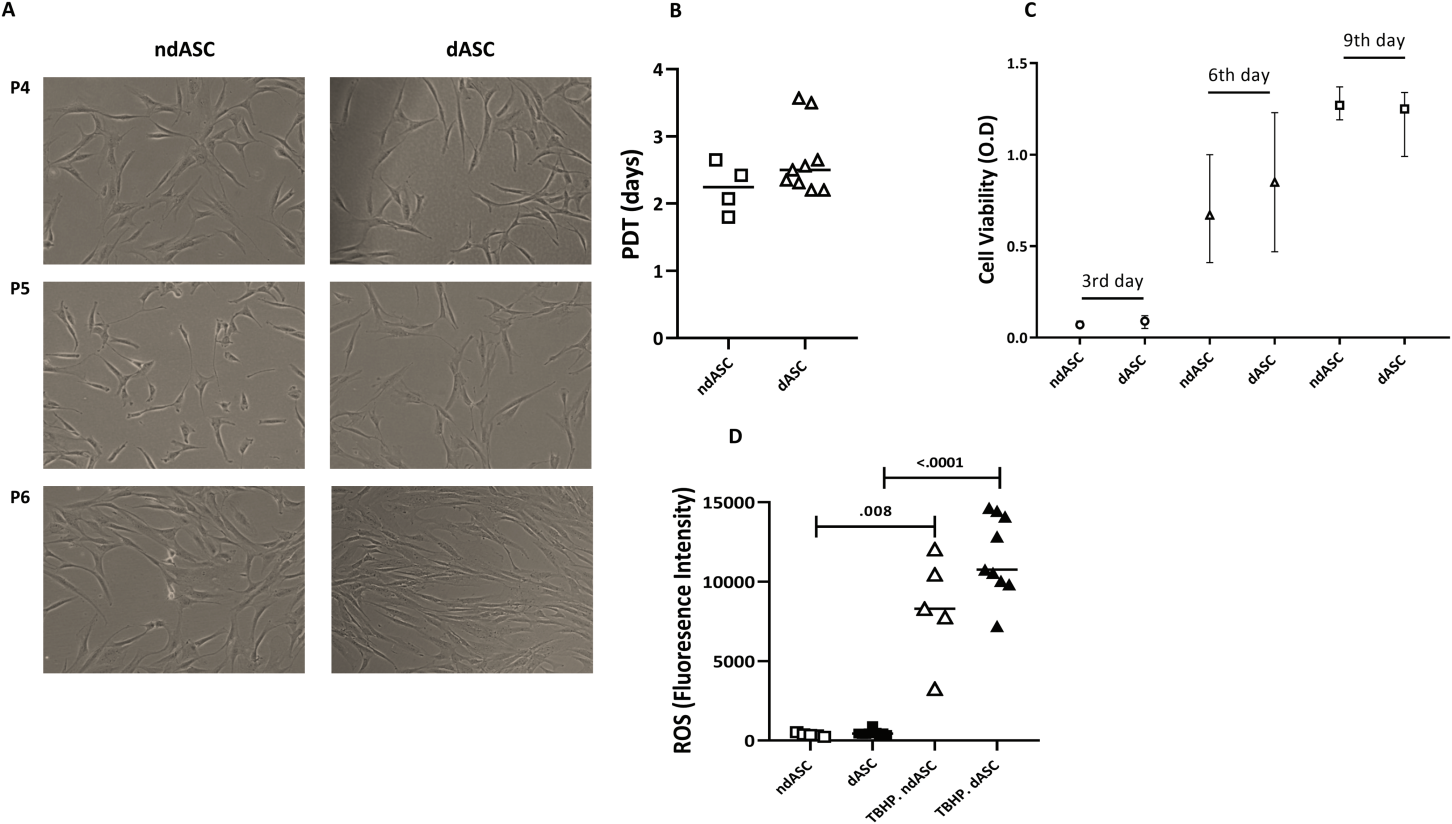
Morphology, proliferation, and ROS levels of ndASCs vs. dASCs. (**A**) ndASCs and dASCs exhibited a typical fibroblast-like morphology at passages ≤5, and both ASC populations started to flatten and striate at P6. (**B**) The dASC proliferation rate was similar to that of ndASCs, as evidenced by comparable median population doubling times (PDTs). (**C**) Data represented as the medians and ranges for a CCK-8 assay demonstrated continuous increases in cell proliferation and viability and a similar proliferative capacity for ndASCs (*n* = 3) vs. dASCs (*n* = 9) over 9 days. (**D**) ROS levels, represented by the fluorescence intensity, generated at a basal level and in TBHP-treated conditions (oxidative stress condition) by ndASCs (*n* = 5) or dASCs (*n* = 9). Each individual dot represents the average of triplicates. The lines in panels B and D represent the medians. The Mann-Whitney test was used for statistical analysis. ndASCs: adipose stem/stromal cells from nondiabetic donors. dASCs: adipose stem/stromal cells from donors with obesity and type 2 diabetes. Abbreviations: ROS, reactive oxygen species; TBHP, tert-butyl hydroperoxide. *P* ≤ .05 was considered significant. Scale bar = 100 μm.

Both ndASCs and dASCs showed the characteristic MSC spindle morphology (Fig. 1A), and their proliferation rates, represented by the PDT, were comparable (Fig. 1B). A CCK-8 assay confirmed the similar proliferation of ndASCs and dASCs (Fig. 1C). Higher levels of basal and stress-responsive ROS were generated by dASCs than by ndASCs, however, not significantly (Fig. 1D). A significant positive correlation was observed between the PDT and basal ROS level (Spearman *r* = 0.85, *P* = .0004).

### ndASCs and dASCs Exhibit Comparable Surface Phenotypes With Minor Differences

ndASCs (*n* = 5) and dASCs (*n* = 9) exhibited the ASC surface markers profile.^32,33^ They were positive (>85%) for CD90, CD73, CD13 CD105, CD44, and CD29 and negative (<2%) for CD14, CD19, CD45RO, CD235a, HLA-DR, CD31, and CD34. dASCs exhibited low to moderate expression of CD146 (2.18%-23.4%), while in ndASCs, the CD146 expression was <1.5%. dASCs and ndASCs showed low median expression (<2 to > 11%) of CD36 and CD54. The median CD54 MFI was significantly higher in ndASCs than in dASCs (Supplementary Table S3).

### Functional Verification

All ndASCs (*n* = 5) differentiated toward osteogenic lineages, as shown by calcium deposition. In contrast, only 2 out of 9 dASC lines showed an osteogenic capacity (Supplementary Fig. S1). ndASCs and dASCs accumulated lipids, as detected by oil red O staining, in adipogenic differentiation medium (Supplementary Fig. S2). The chondrogenic differentiation efficiency varied among the donors, and some ASC lines formed only small amounts of sulfated glycosaminoglycans. The chondrogenic differentiation outcome was not consistent between ndASCs and dASCs (Supplementary Fig. S3).

### ndASCs and dASCs Respond Similarly to Inflammatory Conditions

**Figure 2.**
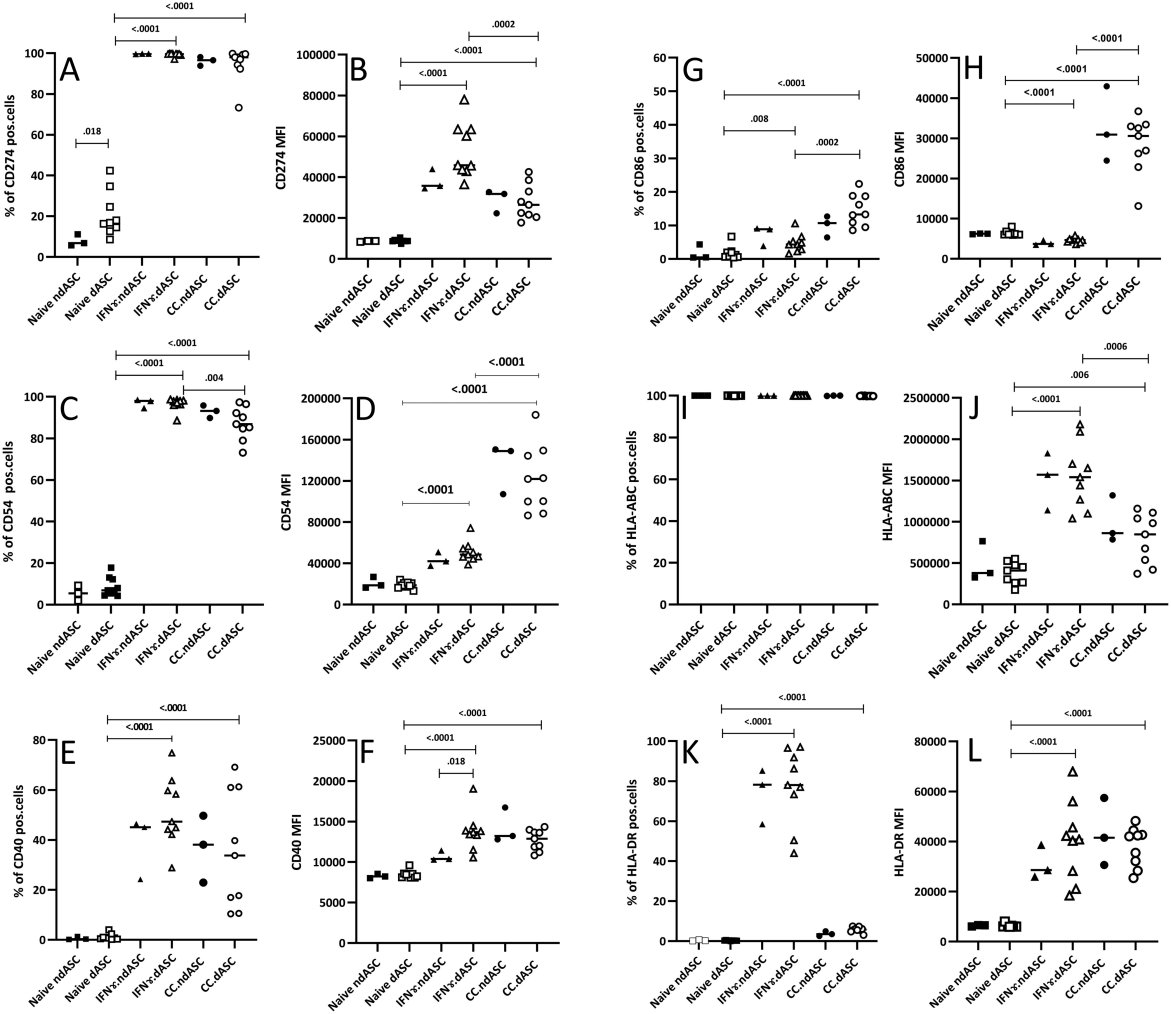
Inflammation-induced expression of immune-related markers by ndASCs and dASCs. ndASCs (*n* = 3) and dASCs (*n* = 9) (at P5) were studied to assess the modulation of immune-related markers in response to treatment with IFN-γ for 48 h or coculture with preactivated PBMCs for 72 h. Both the percentage of positive cells (**A**, **C**, **E**, **G**, **I**, and **K**) and the expression level (MFI) within the positive population (**B**, **D**, **F**, **H**, **J**, and **L**) for each marker are used to present data. Naïve ndASCs and dASCs were negative for CD40 (E), CD86 (G) and HLA-DR (K); however, they expressed CD54 (low) (C), CD274 (low to moderate) (A) and HL-ABC (high) (I). Inflammation highly upregulated the expression of CD274 (A, B) and CD54 (C, D) and moderately upregulated CD40 expression (E, F). The CD54 MFI (D) was greatly increased by coculture with preactivated PBMCs compared to direct IFN-γ priming, indicating the possible implication of this marker in ASC-mediated immunosuppression by facilitating activated lymphocyte binding to ASCs. The HLA-ABC expression level (J) and HLA-DR expression percentage (K) were mainly upregulated after ASC stimulation with IFN-γ; however, the CD86 percentage (G) and MFI (H) were mainly modified after coculture. The horizontal lines represent the medians. The Mann-Whitney test was used for statistical comparisons between groups. Abbreviations: CC, coculture with preactivated PBMCs; dASCs, adipose stem/stromal cells from donors with obesity and type 2 diabetes; MFI, median fluorescence intensity; ndASCs, adipose stem/stromal cells from nondiabetic donors. *P* ≤ .05 was considered significant.

The surface expression of 6 immune-related markers (CD54, CD274, CD40, CD86, HLA-ABC, and HLA-DR) was assessed in ndASCs and dASCs under basal and 2 different inflammatory conditions: coculture with preactivated PBMCs and priming with IFN-γ (Fig. 2). For these markers, only the basal percentage of CD274-positive cells was significantly higher in dASCs than in ndASCs (Fig. 2A). In response to the 2 inflammatory conditions, ndASCs and dASCs upregulated CD54, CD274 (highly), and CD40 (moderately). The CD54 MFI of ndASCs and dASCs was noticeably increased after coculture with preactivated PBMCs compared to priming with IFN-γ (Fig. 2D). The median percentage of CD86^+^ ASCs (Fig. 2G) and the CD86 MFI (Fig. 2H) were mainly affected by coculture with preactivated PBMCs, not IFN-γ priming. Conversely, the expression of HLA molecules was more enhanced by treatment with IFN-γ than by coculture (Fig. 2J, K).

### ndASCs and dASCs Significantly Affect Proliferation and Survival of Stimulated CD4 T Cell

**Figure 3.**
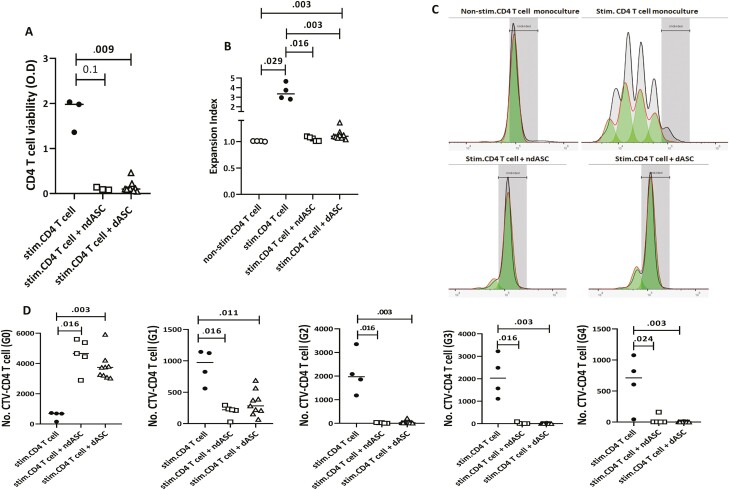
ndASC- and dASC-mediated suppression of CD4 T-cell proliferation. Culture of anti-CD3/CD28-stimulated CD4 T cells in the absence or presence of hASCs for 5 days (1:15, ASC: CD4 T-cell ratio). The data from 2 different experiments, in which 2 CD4 T-cell lines were cultured separately with ndASCs (*n* = 5) or dASCs (*n* = 9), were averaged in the coculture dot plots. The proliferation of viable CD4 T cells was monitored with CCK-8 (**A**) or the cell division tracking dye CTV using FlowJo’s proliferation platform (**B-D**). (A) CCK-8 analysis showed a strongly reduced OD for CD4 T cells stimulated in the presence of ndASCs or dASCs compared to those in monoculture, reflecting the reduced number of viable CD4 T cells in the presence of ASCs. The inhibitory effects of ndASCs and dASCs on CTV-labeled CD4 T-cell proliferation were indicated by the significant reduction in the expansion index (B) and significant arrest of most CD4 T cells in G0 (D) in cocultures. (C) Representative proliferation histograms for viable CD4 T cells in the control nonstimulated, control stimulated, cocultured with ndASCs, and cocultured with dASC groups show the generations in each culture condition. The horizontal lines represent the medians. The Mann-Whitney test was used for statistical comparisons between groups. ndASCs: adipose stem/stromal cells from nondiabetic donors. Abbreviations: CTV, CellTrace violet; dASCs, adipose stem/stromal cells from donors with obesity and type 2 diabetes; OD, optical density; Stim, stimulated. *P* ≤ .05 was considered significant.

**Figure 4.**
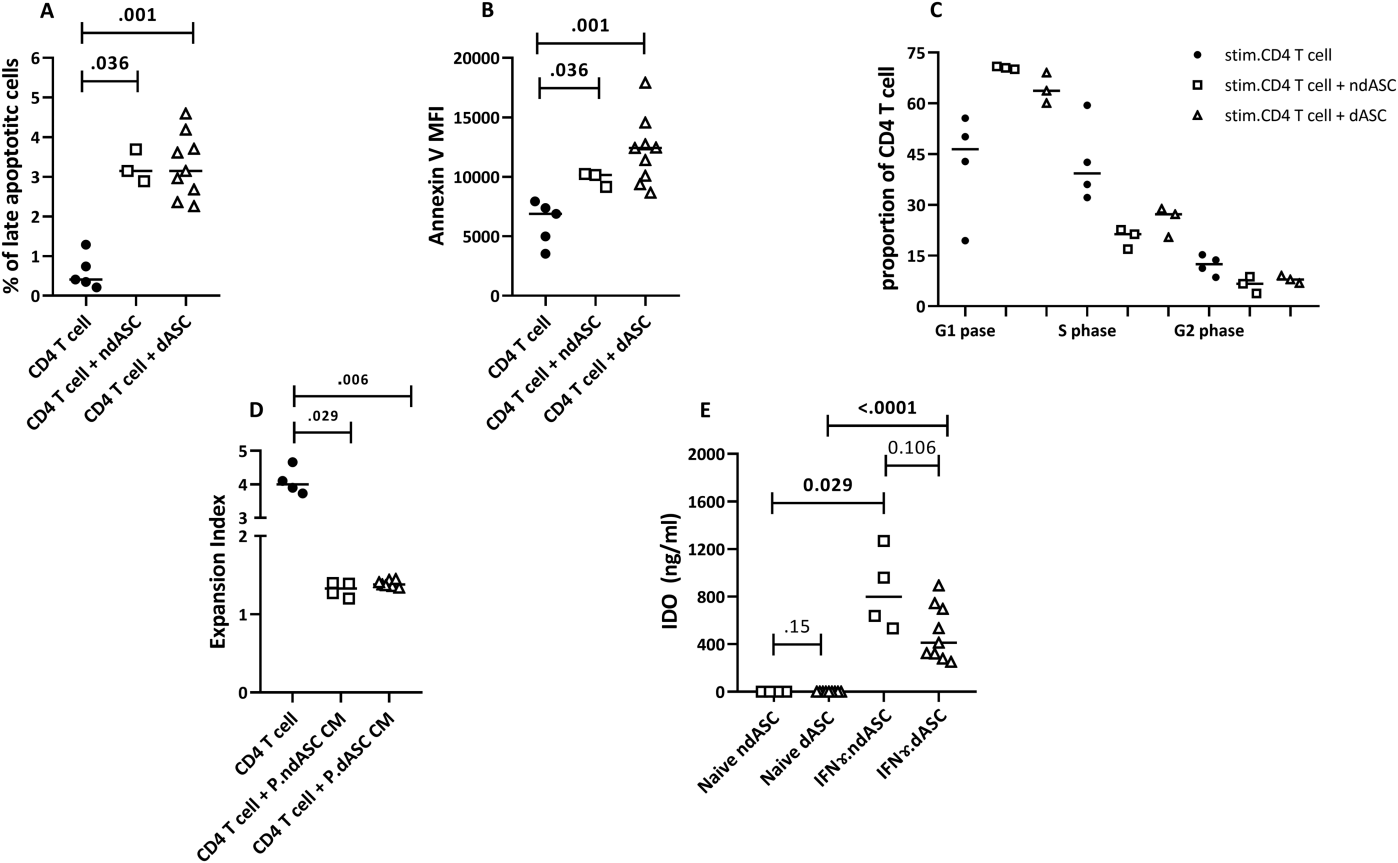
ndASC- and dASC-mediated induction of CD4 T-cell apoptosis. CD4 T cells were stimulated with immobilized anti-CD3/CD28 without or with ndASCs or dASCs (1:15, ASCs: CD4 T cells) for 5 days, and then the cells were stained with annexin V and PI. The data from 2 different experiments, 1 CD4 T-cell line cultured individually with ndASCs (*n* = 3) or dASCs (*n* = 9), were averaged in the coculture dot plots. (**A**) Significant increase in the percentage of late apoptotic cells in the cocultures, associated with an increase in the annexin V MFI (**B**), without statistically significant differences between ndASCs and dASCs. (**C**) Cell cycle phase distribution of stimulated CD4 T cells in coculture with dASCs (*n* = 3) or ndASCs (*n* = 3) relative to a control monoculture. (**D**) Expansion index for anti-CD3/CD28-stimulated CD4^+^ T cells (*n* = 1) cultured in regular medium or the CM of IFN-γ-primed ndASCs (*n* = 4) or dASCs (*n* = 7). (**E**) Strong upregulation of IDO expression in response to IFN-γ priming of ASCs. The horizontal lines represent the medians. The Mann-Whitney test was used for statistical analysis. Abbreviations: dASCs, adipose stem/stromal cells from donors with obesity and type 2 diabetes; ndASCs, adipose stem/stromal cells from nondiabetic donors. *P* ≤ .05 was considered significant.

Stimulation with anti-CD3/CD28 (immobilized form) significantly induced CD4 T-cell division. A marked decrease in the number of viable CD4 T cells was detected in cocultures containing ndASCs or dASCs by CCK-8 analysis, and the expansion index was significantly reduced (Fig. 3A, 3B). In stimulated cocultures, most CD4 T cells were trapped in G0, in contrast to the control stimulated monocultures (Fig. 3C, 3D). The antiproliferative effect of ASCs was associated with a significant increase in the late apoptotic CD4 T-cell proportion (Fig. 4A) and median annexin V MFI (Fig. 4B). According to cell cycle analysis results, the presence of ndASCs or dASCs increased the median proportion of T cells in the G0/G1 phase but decreased those in the S and G2/M phases (Fig. 4C). Importantly, the presence of ASCs significantly (*P* = .016) upregulated the proportion of lymphocytes in the sub-G1 phase, indicating the accumulation of apoptotic cells. Additionally, the CM of IFN-γ-primed ndASCs or dASCs efficiently abrogated CD4 T-cell proliferation (Fig. 4D). We also measured the protein expression of IDO in ndASC and dASC lysates before and after IFN-γ treatment to mimic the inflammatory scenario created by activated T cells.^20,22^ IDO was not considerably expressed by resting ASCs; however, it was upregulated after priming (Fig. 4E).

We tested the potential of ndASCs vs. dASCs to induce an allogeneic T-cell immune response (immunogenicity), but neither ndASCs nor dASCs induced the proliferation of nonstimulated naïve CD4 T cells (Supplementary Fig. S4A) or upregulated HLA-DR expression by the T cells (Supplementary Fig. S4B). The CD25 MFI of CD4 T cells increased after coculture with ndASCs or dASCs (Supplementary Fig. S4C).

### ndASCs and dASCs Regulate CD4 T-cell Activation Markers

**Figure 5.**
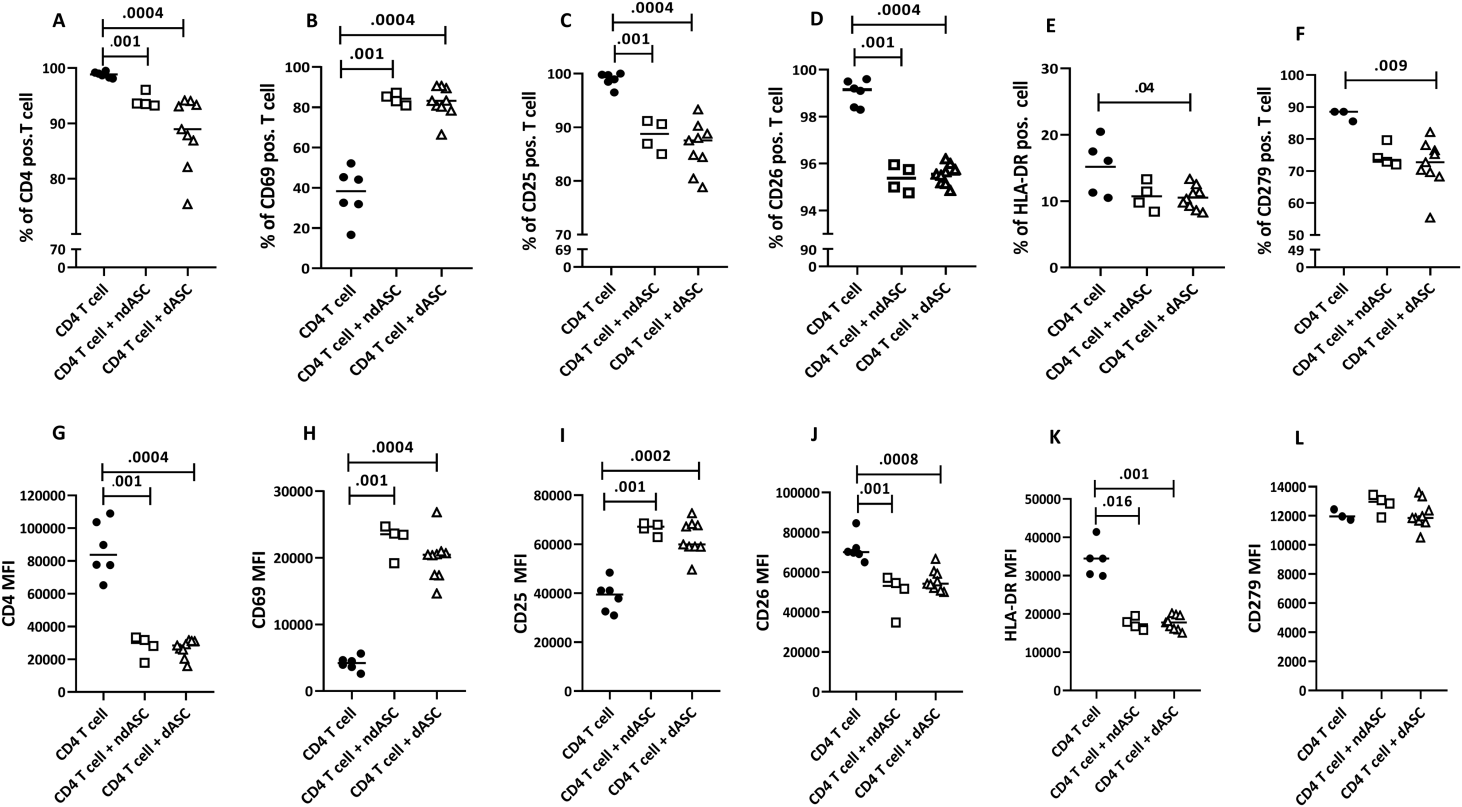
ndASC- and dASC-mediated regulation of the positive-cell percentages (**A-E**) and MFI (**F-J**) of activation markers expressed by the CD4 T cell. CD4 T cells were stimulated with immobilized anti-CD3/CD28 with or without ASCs at a ratio of 1:15 (ASCs: CD4 T cells) for 5 days. Then, the CD4 T cells in stimulated monocultures or cocultures were assessed for T-cell activation, proliferation, and differentiation marker expression. The data from 2 different experiments, in which 2 CD4 T-cell lines were cultured separately with ndASCs (*n* = 5) or dASCs (*n* = 9), were averaged. ndASCs and dASCs were comparably able to modulate the surface activation profile of CD4 T cells. The horizontal lines represent the medians. The Mann-Whitney test was used for statistical analysis,: cluster of differentiation. *P* ≤ 0.05 was considered significant.

Flow cytometric analysis revealed that ndASCs and dASCs slightly, but significantly, reduced the percentage of CD4 positive T cells (Fig. 5A). In contrast, they profoundly reduced the CD4 MFI (Fig. 5G). With anti-CD3/CD28 stimulation, surface expression of CD69, CD25, HLA-DR, CD26, and CD279/PD-1 was induced on CD4 T cells (Supplementary Fig. S6B vs. S6E). ndASCs and dASCs comparably affected the expressions of T-cell activation markers, with no statistically significant differences (Fig. 5). ndASCs and dASCs significantly elevated the expression percentage (Fig. 5B) and level (Fig. 5H) for CD69 on CD4 T cells. Conversely, both ndASCs and dASCs slightly, but significantly, downregulated the CD4^+^ CD25^+^ T-cell percentage (Fig. 5C). Notably, within the CD4^+^ CD25^+^ T-cell population, the CD25 MFI was increased in the presence of ASCs (Fig. 5I). ndASCs and dASCs reduced the CD26 MFI (Fig. 5J) but not the percentage of CD4^+^ CD26^+^ T-cells (Fig. 5D). ndASCs and dASCs decreased the positive percentage and MFI for HLA-DR on CD4 T cells (Fig. 5E, 5K). A significant decrease in the percentage of CD4^+^ CD279^+^ T cells in cocultures was observed (Fig. 5F), while the CD279 MFI within the positive population was not significantly affected (Fig. 5L).

**Figure 6.**
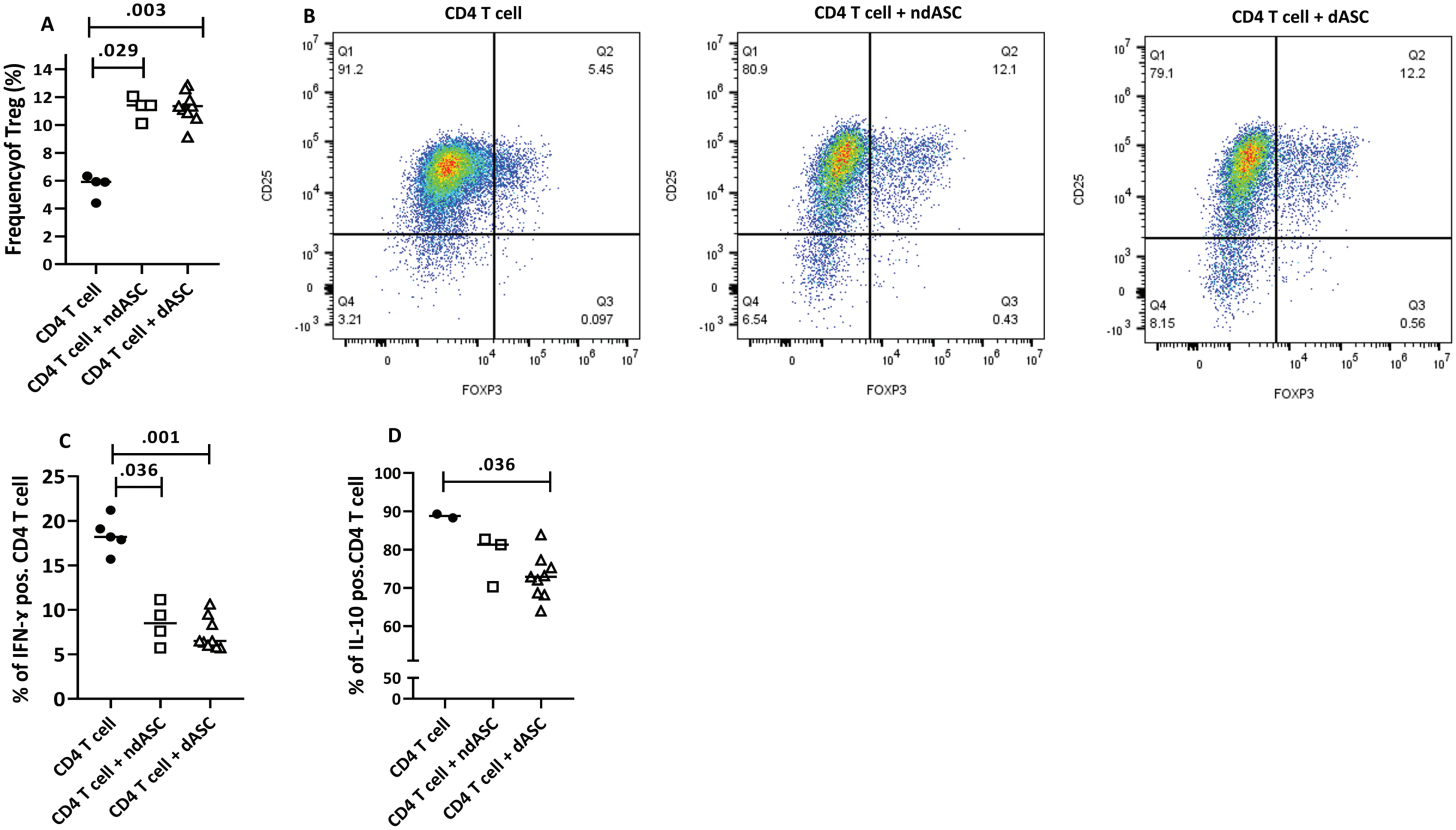
ndASCs and dASCs increase the Treg level and decrease intracellular cytokine expression. CD4 T cells were stimulated with immobilized anti-CD3/CD28 without or with ndASCs (*n* = 3/4) or dASCs (*n* = 4/9) at a ratio of 1:15 for 5 days. Then, the CD4 T cells in the stimulated monoculture and cocultures were assessed to determine the levels of Tregs, IFN-γ and IL-10 via intracellular staining, as described in the Methods. (**A**) and (**B**) show significant upregulation of the level of Tregs, as represented by the increase in the frequency of CD4^+^ CD25^+^ FOXP3^+^ T cells (Q2) by approximately 1.9-fold in cocultures containing either ndASCs or dASCs. The gating strategy for (B) was lymphocyte gating based on FSC and SSC, and viable CD4 T cells were gated and investigated for double-positive CD25 and FOXP3 staining. Analysis of intracellular IFN-γ and IL-10 expression by CD4 T cells showed a considerable reduction for IFN-γ (**C**) and a smaller reduction for IL-10 (**D**) in the presence of ASCs. The results from 2 experiments were averaged and considered in the coculture dot plots and for the IFN-γ and Treg analyses; however, only one investigation was conducted for IL-10. The horizontal lines represent the medians. The Mann-Whitney test was used for statistical analysis. dASCs, adipose stem/stromal cells from donors with obesity and type 2 diabetes; ndASCs, adipose stem/stromal cells from nondiabetic donors; CD, cluster of differentiation. *P* ≤ .05 was considered significant.

### ndASCs and dASCs Significantly Increase Tregs Levels and Decrease Intracellular IFN-γ and IL-10 Expression in T Cells

The presence of ndASCs or dASCs significantly increased the median frequency of Tregs (CD4^+^ CD25^high^ FOXP3^+^). No statistically significant difference in the Tregs frequency was detected between ndASCs and dASCs (*P* = .8) (Fig. 6A). Significant reduction in the percentages of T cells expressing intracellular IFN-γ (Fig. 6C) or IL-10 (Fig. 6D) by both ASC types was detected.

### ndASCs and dASCs Upregulate PGE2 but not Anti-inflammatory Cytokines in Cocultures Containing CD4 T Cells

**Figure 7.**
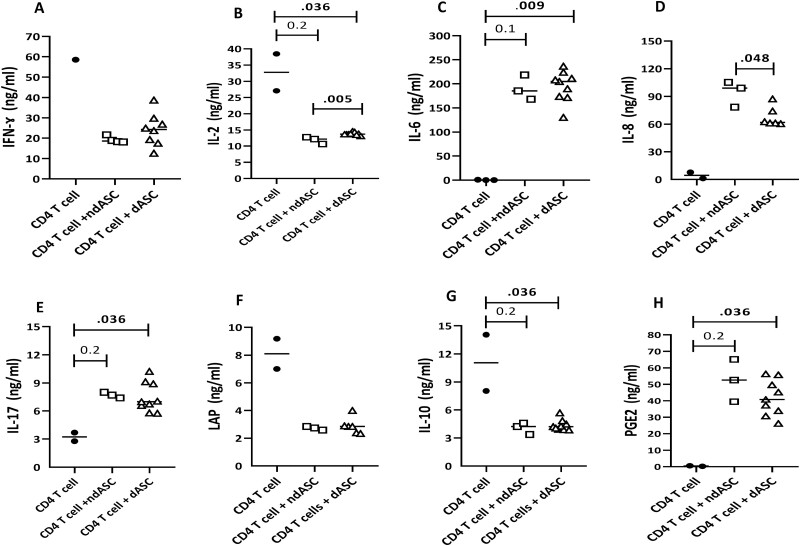
Modulation of CD4 T-cell cytokine secretion by ndASCs and dASCs. For detection of cytokine levels in direct cocultures containing ndASCs (*n* = 3/4) or dASCs (*n* = 6-9) and anti-CD3/CD28-stimulated CD4 T cells (*n* = 2), conditioned media/supernatants were collected on day 5 and stored at –80 °C, and then they were analyzed by ELISA to assess 8 soluble mediators. The results from 2 different CD4 T-cell lines were averaged in the coculture dot plots, except for IFN-γ, for which the results of only one CD4 T-cell line are shown. (**A**) IFN-γ, (**B**) IL-2, (**C**) IL-6, (**D**) IL-8, (**E**) IL-17, (**F**) LAP, (**G**) IL-10, and (**H**) PGE2. The horizontal lines represent the medians. The Mann-Whitney test was used for statistical analysis. Abbreviations: CC, coculture; dASCs, adipose stem/stromal cells from donors with obesity and type 2 diabetes; LAP, latent form of TGF-β1; ndASCs, adipose stem/stromal cells from nondiabetic donors. *P* ≤ .05 was considered significant.

Modulation of cytokines produced by CD4 T helper (Th) cells including the Th1, Th17, and Tregs by ASCs was detected via ELISA (Fig. 7). IFN-γ (Th1) expression was suppressed in the presence of ndASCs or dASCs. Variations in the IFN-γ concentration in response to anti-CD3/CD28 stimulation and ASC-mediated suppression were noticed between the 2 tested CD4 T-cell lines, and only the results of one line are shown (Fig. 7A). IL-2 was suppressed by ASCs, with a slightly but significantly stronger inhibitory effect seen in ndASC cocultures than in dASC ones (Fig. 7B). Conversely, the concentrations of IL-6 (Fig. 7C), IL-8 (Fig. 7D), and IL-17A (Th17) (Fig. 7E) were higher in the ndASC and dASC cocultures than in CD4 T-cell monoculture. Among these cytokines, only IL-8 exhibited a significant difference between the ndASC and dASC cocultures (Fig. 7D). The presence of ndASCs or dASCs in a culture containing stimulated CD4 T cells comparably suppressed the secretion of the Tregs mediators TGF-β1 (LAP) (Fig. 7F) and IL-10 (Fig. 7G) but induced that of PGE2 (Fig. 7H). Considerable levels of IL-6 and TGF-β1 and lower levels of IL-8 and PGE2 were detected in the supernatants of ASCs cultured separately, with nonsignificant differences between ndASCs and dASCs (Supplementary Fig. S8). Basal secretion of IFN-γ and IL-17 by ASCs was not detectable under the applied analytical conditions, and basal secretion of IL-2 and IL-10 by ASCs was not explored.

### Significant Effects of BMI on ASC Immunomodulatory Activity

According to statistical correlation analyses, a high BMI was found to significantly decrease the ASC immunomodulatory potential. There were significant negative correlations between BMI and the basal ASC expression levels of the surface immunomodulator CD54 (Pearson *r* = −0.77, *P* = .001) and the secreted chemokine IL-8 (Spearman *r*= **−**0.82, *P* = .018). A higher BMI significantly affected the ASC potential to decrease IL-2 (Pearson *r* = 0.7, *P* = .017) and promote IL-8 (Pearson *r*= **−**0.84, *P* = .001) secretion in coculture. A higher BMI also significantly correlated with a higher percentage of CD146^+^ ASCs (Spearman *r* = 0.69, *P* = .008), and ASCs with higher CD146 expression had a significantly lower potential to express IDO under inflammatory conditions (Pearson *r*= −0.62, *P* = .025). All correlations are shown in Supplementary Table S4. All *P* values reported in the study from the statistical analyses are illustrated in Supporting File 2.

## Discussion

Donor characteristics such as BMI and health status may affect ASC therapy. Controversial data have been published concerning the effect of T2D and obesity on ASC biological and functional characteristics.^4,28-30,35-40^ Our research is one of the few studies that addressed the immunoregulatory functions of ASCs from T2D donors.^4,28,41^ To our knowledge, this is the first study to explore the effects of T2D and obesity on ASCs’ modulatory functions on purified CD4 T cells. Previous studies have used either Jurkat T lymphocytes,^4^ or whole PBMCs stimulated in mixed lymphocyte reaction,^4,28^ or with a mitogen,^41^ for studying the dASCs’ immunoregulatory functions. In the present study and under mock inflammatory conditions, the expressions of the immunomodulators; CD54, CD274, and IDO were significantly upregulated in ASCs, and no significant differences were noted between dASCs and ndASCs. dASCs significantly suppressed CD4 T-cell proliferation and demonstrated a significantly reduced ability to decrease IL-2 production and to increase IL-8 levels in cocultures. Furthermore, the attenuated potentials were correlated considerably with growing BMI.

Previous reports on the effects of obesity and T2D on ASC proliferation^29,30,35,42^ and antioxidative properties^30^ have been contradictory. In our study, obesity and T2D did not detrimentally impact the proliferation rate of ASCs, although the PDT was slightly longer for dASCs than for ndASCs. Furthermore, we did not find significant differences in the levels of ROS generated by dASCs and ndASCs under basal or oxidative stress conditions, even though dASCs generated higher levels of ROS than ndASCs, suggesting a lower antioxidative potential for the former population. Interestingly, we detected a significant positive correlation between the basal ROS level and PDT (Spearman *r* = 0.85, *P* = .0004). In line with this, an elevated oxidative stress level in ASCs, from donors with T2D, associated with mitochondrial dysfunction and a senescent phenotype has been reported.^30^

Both ndASCs and dASCs complied with the surface marker criteria set by the ISCT^32^ and IFATS^33^ for MSCs, supporting earlier data.^4,28,29^ The expression of the pericytic marker CD146 has been reported to be low (>10%),^43^ or undetectable^44^ in ASCs from nondiabetic donors, while inflammation has been shown to increase its expression.^43^ This may explain our results, in which CD146 expression was detected on dASCs, not on ndASCs. This contrasts with our previous finding suggesting that the percentage of CD146^+^ ASCs and their proangiogenic capacity are significantly higher in ASCs derived from leaner than from heavier donors.^35^ The mesodermal differentiation of dASCs vs. ndASCs was studied and the results have been briefly discussed in the Supplementary File 1.

Naïve ndASCs and dASCs exhibited the immunological profile CD40^neg^ CD86^neg^ HLA-DR^neg^ HLA-ABC^high^ CD274^low to moderate^ CD54^low^. Among the studied markers, only CD274/PD-L1 exhibited significantly higher expression on naïve dASCs than on ndASCs. This is in line with a previous report showing that CD274 expression was significantly higher on ASCs derived from obese donors than on those from lean donors.^45^ This finding may correlate with the enhanced IFN expression in inflamed, obese AT, inducing CD274 expression on AT cellular components.^45^ We detected a significant negative correlation between the CD54 MFI and BMI, supporting an earlier study demonstrating reduced CD54 expression in ASCs from obese donors compared to those from lean donors.^42^ Inflammation is a prerequisite for ASCs to exert immunosuppressive functions via induced expression of paracrine factors and surface molecules such as CD54/ICAM^16^ and CD274/PD-L1.^17,46^ Our study revealed that IFN-γ-primed ndASCs or dASCs, or those cocultured with preactivated PBMCs, reproducibly induced CD40 to reach moderate expression and upregulated CD274 and CD54 to high expression. In both inflammatory conditions, the HLA-DR (HLA-class I) and HLA-ABC (HLA-class I) expression levels increased, with a more potent effect observed for direct IFN-γ treatment. We did not find significant differences in the inflammation-induced expression of immune-related markers between ndASCs and dASCs, suggesting that they could exert comparable immunosuppressive functions.

The immunotolerant profile, indicated by low expression/lack of HLA-DR and the costimulatory molecules CD40 and CD86, which are required for full activation of T lymphocytes,^47^ may contribute to the hypoimmunogenic nature of ASCs.^23,48^ In the present study, neither ndASCs nor dASCs induced the proliferation of allogeneic naïve CD4^+^ CD25^−^ T cells or HLA-DR expression. In contrast, previous studies have illustrated that ASCs have the potential to induce the proliferation of the resting allogeneic CD4 T-cell fraction in PBMCs, suggesting that ASCs are not intrinsically immunoprivileged.^49,50^

In line with previous reports, this study demonstrates the antiproliferative^13,18,20,51,52^ and proapoptotic^17,52^ effects of ASCs on T lymphocytes. Both ndASCs and dASCs significantly inhibited proliferation and induced apoptosis in anti-CD3/CD28-stimulated CD4 T cells. Earlier studies showed a compromised antiproliferative effect for dASCs on PBMCs in a 2-way mixed lymphocyte reaction^28^ and dASC-derived CM on phytohemagglutinin-stimulated T cells.^4^ In our study, there was a trend toward more potent suppression by ndASCs, but the result was not statistically significant. Notably, in the current study, the CM of IFN-γ-primed ndASCs and dASCs significantly abrogated CD4 T-cell proliferation, consistent with previous results;^22^ this suggested the immunotherapeutic efficacy of soluble factors and the possibility of a dASC-free therapy. Priming ASCs with IFN-γ and measuring the level of IDO, the tryptophan-catabolizing enzyme that leads to inhibition of T-cell proliferation, may provide a cost-effective and reproducible method for predicting MSC potency.^53^ In the present study, ndASCs exhibited a higher median IDO concentration than dASCs after IFN-γ treatment, which might explain the greater antiproliferative effect of ndASCs on stimulated CD4 T cells, although the difference was nonsignificant.

To study how T2D and obesity affect the capacity of ASCs to modulate T-cell activation, CD4 T cells in monocultures and cocultures were analyzed for the expression of CD4 and well-known T-cell activation markers, including CD25, CD279, CD26, HLA-DR, and CD69.^54-58^ In our study, ndASCs and dASCs profoundly reduced the expression level of the key receptor CD4 on T cells, suggesting significant inhibition of CD4 T-cell activation by ASCs, as reported previously.^51^

ndASCs and dASCs similarly modulated the expression of activation markers on T cells. ASCs have previously been reported to upregulate CD69,^14,15,53,59,60^ which corresponds to our results, but the opposite effect has also been reported.^61^ We showed a small but significant decrease in the percentage of CD4 T cells expressing CD25 (IL-2 receptor α subunit) in the presence of ASCs, which might have resulted in the reduction in IL-2 signaling and CD4 T-cell proliferation, as previously reported.^16^ CD25 downregulation was associated with a significant reduction in the expression of the coinhibitory molecule CD279/PD-1, as has been previously reported.^62^ However, we detected a significant increase in the CD25 MFI, which might be correlated with the observed increase in the Tregs proportion in cocultures, as Tregs express higher levels of CD25 than effector cells to support their survival.^63^ Importantly, we reported a profound increase in nonproliferating CD4^+^ CD25^+^ CD69^+^ T cells in the presence of ASCs, suggesting T cells with regulatory properties, as previously described.^64^ In our study, the CD26 expression level was significantly reduced in cocultures containing ndASCs or dASCs, and this effect was significantly correlated with the inhibition of CD4 T-cell proliferation by ASCs, supporting previous data.^15^ In our study, the expression of the late activation marker HLA-DR on T cells, which is involved in the “graft-versus-host” reaction,^59^ was significantly compromised by ndASCs and dASCs, which is in line with previous reports.^59,60^

One of the suggested factors leading to immunomodulatory activity dysfunction in ASCs from donors with T2D and obesity is their significantly lower expression of basal TGF-β1 than ASCs from lean, nondiabetic donors.^4,30^ This difference in TGF-β1 expression was not detected in our study and that may contribute to the maintained immunosuppressive potential of dASCs. We addressed the potential of ndASCs and dASCs to modulate the polarization of anti-CD3/CD28-stimulated cells into Th subsets including Th1 cells, Th17 cells and Tregs. Consistent with the well-known ASC-mediated expansion or induction of Tregs,^25,65^ in our study, both ndASCs and dASCs increased the percentage of classical Tregs (CD4^+^ CD25^+^ FOXP3^+^) in stimulated cocultures. We found a negligible level of proliferating cells in stimulated CD4^+^CD25^high^ cells in the presence of ASCs, suggesting that ASCs in cocultures did not promote Tregs expansion. Cell-cell contact and release of PGE2, TGF-β1,^66^ IL-6,^49,64^ PD-L1,^67^ and/or IDO^68^ are among the crucial mechanisms underlying the MSC-mediated induction of Tregs.

Contrary to expectations, ndASCs or dASCs, in our coculture setting, significantly reduced the TGF-β1 and IL-10 concentrations detected in the coculture supernatants. Inconsistent results about the TGF-β1 level in the coculture of ASCs and T cells/PBMCs, relative to control immune cells monoculture, have been earlier illustrated.^14,18,65^ ASCs have been reported either not to change TGF-β1 concentration in coculture with activated T cells,^65^ or they modulated/increased its level in coculture with activated PBMCs/ T cells.^14,18^ TGF-β1 regulates inflammatory responses, and the survival-promoting effect of TGF-β1 on anti-CD3/CD28-stimulated naïve CD4^+69^ or CD4^+^CD44^high70^ T cells has been shown previously. Based on such data, we speculated that the TGF-β1 decrease observed in our cocultures complies with the enhanced apoptosis and decreased viability of CD4 T cells. In concordance with our ELISA results, intracellular detection of IL-10 by flow cytometry revealed a decrease in the presence of ndASCs or dASCs. The lack of an increase in the anti-inflammatory cytokine IL-10 during the Tregs-mediated suppression of alloactivated effector cells has been attributed to its consumption by Tregs via an autocrine IL-10 signaling,^71^ and the same scenario might be present in the current coculture setting.

We detected a significant increase in the secretion of one of the main Th17 cytokine IL-17A^72,73^ in cocultures of ASCs and stimulated CD4 T cells, suggesting Th17-cell differentiation^74,75^ or potentially expansion of a pre-existing Th17-cell population^76^ in the cocultures. The presence of CD4^+^FOXP3^+^ T cells producing IL-17A with immunosuppressive activity, as previously reported,^77^ could not be excluded in the present study. The MSC-mediated induction of Th17 cells has been reported to be concomitant with IL-6^74,76^ or PGE2^75,78^ upregulation. We observed a marked increase in PGE2 and IL-6 secretion when stimulated CD4 T cells and ndASCs or dASCs were cocultured. The immunosuppressive effect mediated by ASCs in our study might be due to the considerable increases in the levels of PGE2,^13,23^ and IL-6,^64,79^ as previously documented. The dual role of the pleiotropic cytokine, IL-6 as an immunosuppressive mediator,^49,64,79,80^ or immunosupportive one,^81,82^ in MSC immunoregulatory functions, has been reported and it is a context-dependent.

In our study, ndASCs or dASCs significantly reduced IFN-γ and IL-2 levels but increased the IL-8 concentration in the coculture supernatant. In support of the ELISA results, flow cytometric analysis of intracellular IFN-γ produced similar results. IFN-γ is one of the functional components of Th1 cells,^72,73^ and the percentage of T cells expressing IFN-γ in anti-CD3/CD28-stimulated PBMCs has been reported to be significantly decreased by ASCs.^17,51^ ndASCs were able to suppress IL-2 levels significantly more strongly than dASCs in cocultures containing CD4 T cells, which might explain the greater antiproliferative effect on CD4 T cells observed with ndASCs. In previous studies, this effect was attributed to the MSC-induced Tregs-mediated deprivation of IL-2^64^ or secretion of PD-1 ligands.^62^ In our study, the basal CD54 expression (CD54 MFI) in ASCs was positively correlated with their antiproliferative effect on T cells and their downregulatory effect on IL-2 levels in cocultures. The level of the chemokine IL-8 was strongly increased in the CD4 T-cell cocultures containing ndASCs or dASCs, with a significantly more potent effect observed with the former cell type. Recently, it has been reported that stimulated CD4 T cells can upregulate CD40 on ASCs and trigger IL-8 production by ASCs in a CD40 ligand-dependent manner, enabling ASCs to perform crosstalk with immune cells in an inflammatory context.^83^

The small sample size of the nondiabetic with normal weight and T2D, obese groups is a study limitation, and larger study groups may be needed to confirm our results. The wide disparity between the BMI for nondiabetic and diabetic donors is a potential study limitation that needs to be considered in future studies. Another limitation of this study could be the significant difference in age between the enrolled diabetic, obese donors (median age 57), and nondiabetic with normal weight donors (median age 42). A larger prospective cohort of diabetic donors may be needed to determine whether age is an important variable that may have biased our findings. However, our findings of the maintained immunomodulatory features of dASCs agree with the results of a previous study on age-matched diabetic and nondiabetic donors.^41^ It has been reported that metformin, a diabetic drug, improves the stemness,^84^ and immunomodulatory potential,^85^ of nondiabetic ASCs. The diabetic donors in the present study were administered different therapeutic regimens for T2D and the unknown effect(s) of medications on ASC functions could be a study limitation that needs to be taken into account in the future. Similarly, unknown donor ethnicity might also be a possible limitation in this study. Additionally, further experiments are needed to unravel the mechanism underlying the ASC-induced generation of Tregs and their immunosuppressive functionality as well as to explore whether the IL-17A increase is correlated with pro-inflammatory or immunosuppressive effects. Moreover, it would be interesting to study the effect of T cells from diabetic donors on ASC immunomodulation capacity.

## Conclusion

Our results indicate that ASCs derived from donors with T2D and obesity can be expanded in vitro and are sensitive to the inflammatory environment, similar to ASCs derived from nondiabetic individuals. The ndASC- and dASC-mediated inhibition of CD4 T-cell proliferation was associated with CD4 T-cell apoptosis. In addition, ndASCs and dASCs comparably modulated the expression of CD4 T-cell activation markers, such as CD69, CD25, CD26, HLA-DR, and CD279, and the secretion of effector cytokines, such as IFN-γ, IL-10, and IL-17A. Both ndASCs and dASCs also significantly increased the Tregs frequency. BMI significantly affected the immunoregulatory properties of ASCs, as shown by the reduced abilities of dASCs to decrease IL-2 production and increase IL-8 levels in cocultures containing CD4 T cells. Such attenuated potentials were significantly correlated with increasing BMI. IFN-γ priming significantly potentiated the immunosuppressive functions of dASCs and ndASCs.

## Supplementary Material

sxad021_suppl_Supplementary_File_S1

sxad021_suppl_Supplementary_File_S2

sxad021_suppl_Supplementary_Material

## Data Availability

The datasets generated and/or analyzed during the current study are available from the corresponding author on reasonable request.
