# Correction: Immunomodulatory Functions of Adipose Mesenchymal Stromal/Stem Cell Derived From Donors With Type 2 Diabetes and Obesity on CD4 T Cells

**DOI:** 10.1093/stmcls/sxad043

**Published:** 2023-06-13

This is a correction to: Marwa Mahmoud and others, Immunomodulatory Functions of Adipose Mesenchymal Stromal/Stem Cell Derived From Donors With Type 2 Diabetes and Obesity on CD4 T Cells, Stem Cells, Volume 41, Issue 5, May 2023, Pages 505–519, https://doi.org/10.1093/stmcls/sxad021

In the originally published version of this manuscript, there were errors in references to two figures Under subheading ndASCs and dASCs Significantly Affect Proliferation and Survival of Stimulated CD4 T Cell, section following should read: The antiproliferative effect of ASCs was associated with a significant increase in the late apoptotic CD4 T-cell proportion (Fig. 4A) and median annexin V MFI (Fig. 4B). According to cell cycle analysis results, the presence of ndASCs or dASCs increased the median proportion of T cells in the G0/G1 phase but decreased those in the S and G2/M phases (Fig. 4C). Importantly, the presence of ASCs significantly (P =.016) upregulated the proportion of lymphocytes in the sub-G1 phase, indicating the accumulation of apoptotic cells. Additionally, the CM of IFN-γ-primed ndASCs or dASCs efficiently abrogated CD4 T-cell proliferation (Fig. 4D). We also measured the protein expression of IDO in ndASC and dASC lysates before and after IFN-γ treatment to mimic the inflammatory scenario created by activated T cells.20,22 IDO was not considerably expressed by resting ASCs; however, it was upregulated after priming (Fig. 4E).” instead of reading Fig. 3A-E throughout.

Under subheading ndASCs and dASCs Regulate CD4 T-cell Activation Markers, the fifth sentence should read: “Notably, within the CD4^+^ CD25^+^ T-cell population, the CD25 MFI was increased in the presence of ASCs (Fig. 5).” instead of: “Notably, within the CD4^+^ CD25^+^ T-cell population, the CD25 MFI was increased in the presence of ASCs (Fig. 6).”

These errors have been corrected in the article.

Addendum to the above 1 July 2023: There was an error in the above correction. Under subheading ndASCs and dASCs Regulate CD4 T-cell Activation Markers, the fifth sentence should read: “Notably, within the CD4+ CD25+ T-cell population, the CD25 MFI was increased in the presence of ASCs (Fig. 5I).”The following errors were erroneously omitted:Under subheading ndASCs and dASCs Significantly Increase Tregs Levels and Decrease Intracellular IFN-γ and IL-10 Expression in T Cells, the third sentence should read: “Significant reduction in the percentages of T cells expressing intracellular IFN-γ (Fig. 6C) or IL-10 (Fig. 6D) by both ASC types was detected.” instead of: “Significant reduction in the percentages of T cells expressing intracellular IFN-γ (Fig. 5C) or IL-10 (Fig. 5D) by both ASC types was detected.”These errors are corrected in the article.

