# Supplementary material for: Immunomodulatory Functions of Adipose Mesenchymal Stromal/Stem Cell Derived From Donors With Type 2 Diabetes and Obesity on CD4 T Cells
Source: Stem Cells. 2023 Mar 22;41(5):505–19. doi: 10.1093/stmcls/sxad021 (PMC10183970; doi:10.1093/stmcls/sxad021)
Supplement: sxad021_suppl_Supplementary_File_S1 [file sxad021_suppl_supplementary_file_s1.pdf]

Methods and Discussion of functional verification of dASCs vs ndASCs

Methods

Osteogenic Differentiation

Differentiation medium composition and culture conditions are described previously [1]. ndASCs or dASCs at passage 5 or 6 were cultured for about 3 weeks in osteogenic differentiation [OD] medium supplemented with bioactive glass S53P4 BaG granules (500-1000µm), which was changed twice a week. Differentiated ASCs were stained with Alizarin Red to study the cellular potential of producing mineralized calcium deposits.

Adipogenic Differentiation

Differentiation medium composition and culture conditions were briefly described [2]. Briefly, 7.5×10^4 ndASCs or dASCs / well were seeded on a 24 well culture plate for 24 hrs then, the growth medium was replaced with differentiation medium (Dulbecco's modified Eagle's medium/Nutrient Mixture F-12 (DMEM/F12) (Gibco), 3% HS (Serana), 100 U/ml penicillin and 100µg/ml streptomycin (Lonza), 100 nM Insulin (Gibco), 1 μM Dexamethasone (Sigma), 0.5 mM 3-isobutyl-1-methylxanthine (IBMX) (Sigma), 1 μM Rosiglitazone (MERCK Millipore), 33 μM Biotin (Sigma) and 17 μM Pantothenate (MERCK)). Induction was performed for 7 days. After that differentiation medium was changed to maintenance medium (DMEM/F12, 3% HS, 100 nM Insulin, 1 μM Dexamethasone, 33 μM Biotin and 17 μM Pantothenate). Duration for maintenance medium was for 7-14 days, the medium was changed in every 4-5 days. During the differentiation process, cells were cultured for about 3 weeks. Differentiated ASCs were stained with Oil Red O and DAPI to study the cellular potential of lipid accumulation. ASCs cultured in control medium (DMEM F/12 + 1% Glutamax with 5% HS, 100 U/ml penicillin and 100µg/ml streptomycin) were used as negative control. Samples were photographed with a fluorescence microscope (Nikon Olympus).

Chondrogenic Differentiation

Differentiation medium composition and culture conditions were briefly described [3]. Briefly, 8×10^4 ndASCs or dASCs were seeded on a 24-well culture plate in a 10-µl volume and were allowed to adhere for 3 hours before the addition of chondrogenic induction medium (DMEM F/12 + 1% Glutamax, 100 U/ml penicillin and 100µg/ml streptomycin, 1x ITS+1 (Sigma),

50µg/ml Ascorbic Acid-2-phosphate (Sigma), 55µg/ml Na-pyruvate (Lonza), 23µg/ml L-proline (Sigma) and 2µg/ml TGF-β1 (Sigma)). Medium was changed twice a week. After 14 days of chondrogenic induction, differentiation was confirmed by using the Alcian blue staining method. For that, ASC pellets were rinsed with DPBS and fixed with 4% PFA. Subsequently, cells were rinsed twice with deionized water and stored in 70% ethanol. Pellets were dehydrated, embedded in paraffin, and sectioned at 4µm thickness. The sections were rehydrated and stained with Alcian blue (Sigma) (pH 1.0) to detect sulfated glycosaminoglycans (GAGs) by using Nuclear Fast Red - aluminium sulfate solution (Sigma). Samples were photographed with microscope scanner (Hamamatsu S60).

Discussion

In the current study, dASCs showed compromised osteogenic differentiation, while maintained adipogenic differentiation. Chondrogenic differentiation was varied among the cell lines of ndASCs and dASCs groups. The effect of obesity or T2D on ASCs differentiation capacity has been studied previously, but the results have been contradictory [4-11]. In comply with our results, ASCs, isolated from donors with obesity [5] or T2D [6], showed impaired osteogenic differentiation and that may be attributed the enhanced inflammation associated with obesity or T2D [7]. Maintained adipogenic [8-10] and chondrogenic [6, 8] differentiation capacities have been previously reported.

References

1. Ojansivu M, Vanhatupa S, Bjorkvik L, Hakkanen H, Kellomaki M, Autio R, et al. Bioactive glass ions as strong enhancers of osteogenic differentiation in human adipose stem cells. *Acta Biomater* 2015;21:190-203.
2. Maenpaa K, Ella V, Mauno J, Kellomaki M, Suuronen R, Ylikomi T, et al. Use of adipose stem cells and polylactide discs for tissue engineering of the temporomandibular joint disc. *J R Soc Interface* 2010;7(42):177-88.
3. Herbers E, Patrikoski M, Wagner A, Jokinen R, Hassinen A, Heinonen S, et al. Preventing White Adipocyte Browning during Differentiation In Vitro: The Effect of Differentiation Protocols on Metabolic and Mitochondrial Phenotypes. *Stem Cells Int* 2022;2022:3308194.
4. Strong AL, Hunter RS, Jones RB, Bowles AC, Dutreil MF, Gaupp D, et al. Obesity inhibits the osteogenic differentiation of human adipose-derived stem cells. *J Transl Med* 2016;14:27.
5. De Girolamo L, Stanco D, Salvatori L, Coroniti G, Arrigoni E, Silecchia G, et al. Stemness and osteogenic and adipogenic potential are differently impaired in subcutaneous and visceral adipose derived stem cells (ASCs) isolated from obese donors. *Int J Immunopathol Pharmacol* 2013; 26(1 Suppl):11-21.
6. Wang L, Zhang L, Liang X, et al. Adipose Tissue-Derived Stem Cells from Type 2 Diabetics Reveal Conservative Alterations in Multidimensional Characteristics. *Int J Stem Cells* 2020; 13(2):268-78.
7. Onate B, Vilahur G, Ferrer-Lorente R, Ybarra J, Diez-Caballero A, Ballesta-Lopez C, et al. The subcutaneous adipose tissue reservoir of functionally active stem cells is reduced in obese patients. *FASEB J* 2012;26(10):4327-36.
8. Aliakbari S, Mohammadi M, Rezaee MA, et al. Impaired immunomodulatory ability of type 2 diabetic adipose-derived mesenchymal stem cells in regulation of inflammatory condition in mixed leukocyte reaction. *EXCLI J* 2019; 18:852-65.
9. Dentelli P, Barale C, Togliatto G, Trombetta A, Olgasi C, Gili M, et al. A diabetic milieu promotes OCT4 and NANOG production in human visceral-derived adipose stem cells. *Diabetologia* 2013;56(1):173-84.
10. Minteer DM, Young MT, Lin YC, Over PJ, Rubin JP, Gerlach JC, et al. Analysis of type II diabetes mellitus adipose-derived stem cells for tissue engineering applications. J *Tissue Eng* 2015;6:2041731415579215.
11. Juntunen M, Heinonen S, Huhtala H, et al. Evaluation of the effect of donor weight on adipose stromal/stem cell characteristics by using weight-discordant monozygotic twin pairs. *Stem Cell Res Ther* 2021; 12(1):516.
