# Supplementary material for: Immunomodulatory Functions of Adipose Mesenchymal Stromal/Stem Cell Derived From Donors With Type 2 Diabetes and Obesity on CD4 T Cells
Source: Stem Cells. 2023 Mar 22;41(5):505–19. doi: 10.1093/stmcls/sxad021 (PMC10183970; doi:10.1093/stmcls/sxad021)
Supplement: sxad021_suppl_Supplementary_File_S2 [file sxad021_suppl_supplementary_file_s2.pdf]

Supporting File 2 includes P values reported from all statistical analyses

Group	Key
Non diabetic Donors with normal weight	**A**
Diabetic Donors with obesity	**B**
Basal/naive ndASCs	**C**
Basal/naïve dASCs	**D**
IFN-ɣ treated ndASCs	**E**
IFN-ɣ treated dASCs	**F**
TBHP-treated ndASCs	**G**
TBHP-treated dASCs	**H**
CC. ndASCs	**I**
CC.dASCs	**J**
stim.CD4 T cells	**K**
stim.CD4 T cells + ndASCs	**L**
stim.CD4 T cells + dASCs	**M**

Variables	A vs B *(P value)*	C vs D *(P value)*	C vs E *(P value)*	D vs F *(P value)*	E vs F *(P value)*	G vs H *(P value)*	C vs G *(P value)*	D vs H *(P value)*			
**Age**	**0.0115**										
**BMI**	**0.0010**										
**ASC proliferation**											
**1. PDT**		0.269									
**2. CCK8**											
**2.1. 1st**		0.55									
**2.2. 2nd**		0.6									
**2.3. 3rd**		0.56									
**ASC ROS level**		0.119				0.112	**0.008**	**<0.0001**			
**ASC-IDO**		0.146	**0.029**	**<0.0001**	0.106						
**ASC immunophenotype**											
CD90%		0.4									
CD105 %		0.09									
CD73 %		>0.999									
CD44 %		0.25									
CD29 %		0.8									
CD13 %		0.45									
CD14 %		0.97									
CD19 %		0.65									
CD45RO %		0.38									
HLA-DR %		0.97									
CD31 %		0.97									
CD34 %		0.25									
CD235A %		0.87									
CD146 %		**0.001**									
CD36 %		0.8									
CD54 %		0.9									
CD90 MFI		0.7									
CD105 MFI		0.68									
CD73 MFI		0.24									
CD44 MFI		0.35									
CD29 MFI		0.15									

CD13 MFI		0.24									
CD14 MFI		0.09									
CD19 MFI		0.06									
CD45RO MFI		0.18									
HLA-DR MFI		**0.007**									
CD31 MFI		**0.02**									
CD34 MFI		**0.04**									
CD235A MFI		0.09									
CD146 MFI		0.52									
CD36 MFI		0.19									
CD54 MFI		**0.007**									
**Item**	**A vs B** ***(P value)***	**C vs D** ***(P value)***	**C vs E** ***(P value)***	**D vs F** ***(P value)***	**E vs F** ***(P value)***				**I vs J** ***(P value)***	**C vs I** ***(P value)***	**D vs J** ***(P value)***
**Immune-related ASC surface markers**											
**CD40%**		0.455	0.1	**<0.0001**	0.373				0.864	0.1	**<0.0001**
**CD86%**		0.6	0.2	**0.0078**	0.373				0.146	0.1	**<0.0001**
**HLA-DR%**		0.7	0.1	**<0.0001**	0.86				0.063	0.1	**<0.0001**
**HLA-ABC%**		>0.9999	>0.9999	**>0.9999**	>0.9999				0.355	>0.9999	**0.0115**
**CD54%**		0.4	0.1	**<0.0001**	0.97				0.372	0.1	**<0.0001**
**CD274%**		**0.0182**	0.1	**<0.0001**	0.677				0.7	0.1	**<0.0001**
**CD40 MFI**		0.441	0.1	**<0.0001**	0.018				0.482	0.1	**<0.0001**
**CD86 MFI**		0.6	0.1	**<0.0001**	0.1				0.727	0.1	**<0.0001**
**HLA-DR MFI**		0.486	0.1	**<0.0001**	0.372				0.864	0.1	**<0.0001**
**HLA-ABC MFI**		0.6	0.1	**<0.0001**	>0.9999				0.482	0.1	**0.006**
**CD54 MFI**		0.755	0.1	**<0.0001**	0.282				0.373	0.1	**<0.0001**
**CD274 MFI**		0.3	0.1	**<0.0001**	0.06				0.864	0.1	**<0.0001**

Variables	K vs L	K vs M	L vs M
**T Cells Apoptosis**			
Annexin V MFI	0.036	0.001	0.282
Annexin V^+ PI^- % (Early Apoptosis)	0.392	0.438	0.864
Annexin V^+ PI^+% (Late Apoptosis)	**0.036**	**0.001**	>0.9999
Annexin V^- PI^+ % (Dead)	0.786	0.506	0.282
**Cell Cycle Analysis**			
1. G1 phase	0.057	0.057	0.1
2. S Phase	0.057	0.057	0.4
3. G2 phase	0.114	0.114	0.4
**ASCs Effects on T cells Proliferation**			
1. G0	**0.016**	**0.003**	0.24
2. G1	**0.016**	**0.011**	0.364
3. G2	**0.016**	**0.003**	0.346
4. G3	**0.016**	**0.003**	0.898
5. G4	**0.024**	**0.003**	0.873
6. Expansion Index	**0.016**	**0.003**	0.068
7. CCK8 for CD4 T cells viability	0.1	0.009	0.972
**IFN-ɤ Primed ASCs-CM Effects on T cells Proliferation**			
Expansion Index	**0.029**	**0.006**	0.315
**T Cells Surface Activation Markers**			
CD4 %	**0.0095**	**0.0004**	0.106
CD69 %	**0.0095**	**0.0004**	0.825
CD25 %	**0.0095**	**0.0004**	0.414
CD26 %	**0.0095**	**0.0004**	0.629
HLA-DR %	0.191	**0.0420**	0.94
CD279%	**0.0571**	**0.0091**	0.6042

Variables	K vs L	K vs M	L vs M
CD4 MFI	**0.0095**	**0.0004**	0.504
CD69 MFI	**0.0095**	**0.0004**	0.199
CD25 MFI	**0.0095**	**0.0002**	0.312
CD26 MFI	**0.0095**	**0.0008**	0.504
HLA-DR MFI	**0.016**	**0.0010**	0.712
CD279MFI	0.229	0.864	0.199
**Analysis of Intracellular T cells Markers**			
1. Tregs %	**0.029**	**0.003**	0.796
2. IFN-ɤ%	**0.016**	**0.001**	0.578
3. IL-10%	0.2	**0.036**	0.373
**Analysis of Soluble Cytokines and Factors**			
1. IFN-ɤ	---	----	0.376
2. IL-2	0.20	**0.0364**	**0.0045**
3. IL-6	0.10	**0.0091**	0.727
4. IL-8	0.20	**0.0714**	**0.048**
5. IL-17A	0.20	**0.0364**	0.482
6. IL-10	0.20	**0.0364**	0.864
7. LAP/ TGF-β1	0.20	**0.0714**	0.714
8. PGE2	0.20	**0.0364**	0.282

BMI: Body Mass Index, ASCs: Adipose Mesenchymal Stem/Stromal Cells, PDT: Population Doubling Time, CD: Cluster of Differentiation, HLA: Human Leukocyte Antigen, ROS: Reactive oxygen Species, IDO: Indoleamine 2,3-Dioxygenase, %: Percentage, MFI: Median Fluorescence Intensity, CM: conditioned Medium, G0-G4: Division Generations 0-4, Tregs: Regulatory T Cells, IFN-ɤ: Interferon Gamma, IL: Interleukin, TGF-β1: Transforming Growth Factor Beta 1, PGE2: Prostaglandin E2. P≤ 0.05 was considered significant.
