# Supplementary material for: Immunomodulatory Functions of Adipose Mesenchymal Stromal/Stem Cell Derived From Donors With Type 2 Diabetes and Obesity on CD4 T Cells
Source: Stem Cells. 2023 Mar 22;41(5):505–19. doi: 10.1093/stmcls/sxad021 (PMC10183970; doi:10.1093/stmcls/sxad021)
Supplement: sxad021_suppl_Supplementary_Material [file sxad021_suppl_supplementary_material.pdf]

Supplementary Table 1: Characteristics of ASCs donors in the study

ASC line Code	Age	BMI	Sex	Diabetes Medications	Anatomical Location	Race
D1	62	37	Male	Victoza	Abdomen	Finnish
D2	55	48.5	Female	Diformin retard	Abdomen	Finnish
D3	54	43.3	Female	Diformin	Abdomen	Finnish
D4	57	40.4	Female	Diformin 1 g, Victoza, Levemir	Abdomen	Finnish
D5	59	32.6	Female	Diformin 500 mg tab	Abdomen	Finnish
D6	61	50.9	Female	NA	Abdomen	Finnish
D7	57	49.3	Female	Metforem 500 mg	Abdomen	Finnish
D8	50	41.7	Female	Metforem 500 mg	Abdomen	Finnish
D9	51	42.6	Female	Metforem 500 mg	Abdomen	Finnish
ND1	56	25.2	Male	NA	Abdomen	Finnish
ND2	31	24.4	Female	NA	Abdomen	Finnish
ND3	42	22.5	Female	NA	Abdomen	Finnish
ND4	21	26.3	Female	NA	Breast	Finnish
ND5	44	27.4	Female	NA	Abdomen	Finnish

ASC: adipose stem/ stromal cells, D: diabetic, ND: nondiabetic, BMI: body mass index, NA: not applicable

Supplementary Table 2: Antibodies against surface protein markers and manufacturers

Antibody	Surface Antigen	Manufacturer
CD13-BV421	Amino peptidase N (APN)	BD Pharmingen
CD14-APC	Lipopolysaccharide receptor	BD Pharmingen
CD19-APC	B lymphocyte antigen	BD Pharmingen
CD29-FITC	Very late antigen-4 (VLA-4)	Immunotools
CD31-BV421	Platelet endothelial cell adhesion molecule (PECAM-1)	BD Pharmingen
CD34-APC	Hematopoietic progenitor cell antigen	Immunotools
CD36-APC	Platelet glycoprotein 4	BD Pharmingen
CD44-FITC	CD44 antigen (adhesion and migration)	BD Pharmingen
CD45R0-APC	RO isoform of leucocyte common antigen	BD Pharmingen
CD54-FITC	Intracellular adhesion molecule-1 (ICAM-1)	BD Pharmingen
CD73-PE	Lymphocyte-vascular adhesion protein (L-VAP-2) or Ecto 5'-nucleotidase	BD Pharmingen
CD90-APC	Thy-1	BD Pharmingen
CD105-PE	Endoglin	R&D Systems
CD146-BV421	Melanoma Cell adhesion molecule (MCAM)	BD Pharmingen
CD235a-BV421	Glycophorin A	BD Pharmingen
HLA-DR-BV421	Major Histocompatibility Complex Class II (MHC II)	BD Pharmingen
HLA-ABC-APC	Major Histocompatibility Complex Class I (MHC I)	BD Pharmingen

CD40-APC	Costimulatory molecule CD40	BD Pharmingen
CD86-PE	B7-2 (CD28 ligand)	BD Pharmingen
CD274-APC	Programmed death ligand 1 (PD-L1)	BD Pharmingen

CD: cluster of differentiation, BD: Becton, Dickinson, FITC: fluorescein isothiocyanate, APC: allophycocyanin, PE: phycoerythrin, BV: brilliant violet

Supplementary Table 3: Surface marker expression of ndASCs and dASCs.

% positive ASCs	ndASCs (n=5)			dASCs (n=9)			*P value*
	**Median**	**Min**	**Max**	**Median**	**Min**	**Max**	
CD90	100	99.7	100	100	99.9	100	0.4
CD105	100	100	100	99.9	99.9	100	0.09
CD73	100	99.9	100	100	99.9	100	>0.999
CD44	99.7	94	99.9	97.1	92.5	99.9	0.25
CD29	79.8	82.3	98.8	94.9	83.8	99.9	0.8
CD13	99.1	86.5	99.9	99.5	92.3	99.8	0.45
CD14	0.3	0.16	0.4	0.25	0.19	0.47	0.97
CD19	0.2	0.15	0.33	0.22	0.12	0.4	0.65
CD45RO	0.35	0.22	1.83	0.73	0.25	1.72	0.38
HLA-DR	0.3	0.26	0.37	0.29	0.17	0.5	0.97
CD31	0.28	0.19	0.4	0.32	0.077	0.5	0.97
CD34	0.49	0.2	0.76	0.3	0.17	0.63	0.25
CD235A	0.31	0.27	0.44	0.33	0.069	0.66	0.87
CD146	0.7	0.4	1.35	9.79	2.18	23.4	**0.001**
CD36	4.47	3.35	41.3	10.8	1.34	47.3	0.8
CD54	2.04	1.05	9.81	2.99	1.3	5.69	0.9
**MFI**	**ndASCs**			**dASCs**			***P value***

	Median	Min	Max	Median	Min	Max	
CD90	750000	365303	1470000	844000	420000	1770000	0.7
CD105	593000	410000	1260000	589000	419000	893000	0.68
CD73	583000	360245	650000	352789	243040	1100000	0.24
CD44	679000	421000	865000	483000	287241	899000	0.35
CD29	164668	135126	190529	150461	84431	267918	0.15
CD13	211471	186598	325617	159050	104950	307963	0.24
CD14	12721	9904	14823	9444	8645	13797	0.09
CD19	12887	10389	13797	9686	7821	14394	0.06
CD45RO	12929	10455	20681	10624	9121	20544	0.18
HLA-DR	64552	59857	116831	39459	36993	64997	0.007
CD31	62803	54754	111306	40272	37372	64997	0.02
CD34	13355	10864	14023	9904	8069	13797	0.04
CD235A	54195	53275	100338	42817	37372	89531	0.09
CD146	66809	55891	109019	64997	47754	81570	0.52
CD36	56468	53093	97266	51483	24030	83275	0.19
CD54	71565	57248	93317	47591	42382	67733	0.007

ndASCs: ASCs from nondiabetic donors, dASCs: ASCs from patients with T2D and obesity, MFI: median fluorescence intensity

Supplementary Table 4: Significant statistical correlations between studied parameters.

Correlation arms		r	Significance
**BMI**	IL-2 CC	.7	**.017**
	% of CD146+ ASCs	.69	**.008**
	IL-8 CC	-.84	**.001**
	Basal IL-8, produced by resting ASCs	-.81	**.018**
	CD54 MFI	-.77	**.001**
**CD54 MFI**	Basal IL-8	0.83	**0.011**
	G0	.6	**.025**
	CD73 MFI	.58	**.04**
	IL-8	.52	**.19**
	IL-2 CC	-.84	**.0006**
**% of CD146+ ASCs**	CD26 MFI	.55	**.05**
	CD69 MFI	-.7	**.008**
	IFN-ɣ–induced IDO	-0.62	**.025**
**CD13 MFI**	IFN-ɣ CC	-.79	**0.004**
	IL-17 CC	-0.77	**.003**
	CD4 MFI	-.72	**.006**
	CD279%	-.7	**.009**
	CD279 MFI	-.51	**.07**
**CD29 MFI**	IFN-ɣ CC	-.78	**.005**
	IL-17 CC	-.54	**.07**

	G2	-.55	**.045**
	CD4 MFI	-.64	**.017**
	CD25 MFI	-.58	**.037**
	CD279%	-.7	**.008**
	CD69%	-.63	**.022**
**CD44 MFI**	IFN-ɣ CC	-.77	**.005**
	IL-17 CC	-.56	**.048**
	CD279%	-.57	**.04**
**CD73 MFI**	IFN-ɣ CC	-.67	**.028**
	IL-17 CC	-.6	**.06**
	IL-6 CC	-.55	**.07**
	CD279%	-.63	**.03**
**CD105 MFI**	G0	.5	**.06**
	Basal IL-6	-0.56	**.05**
	IL-17	-.5	**.1**
**% of CD274$^+$ ASCs**	G1	.75	**.005**
	IL-2 CC	.52	**.08**
	CD274 MFI.IFN	.64	**.026**
	PGE2 CC	-.54	**.07**
**Basal LAP/ TGF-β1**	PGE2 CC	-.87	**.004**
**Basal IL-8**	IL-2 CC	- .8	**.018**
**Basal PGE2**	CD73 MFI	-.52	**.055**

	LAP CC	-.68	**.048**
	CD26%	-.62	**.02**
	CD26 MFI	.53	**.06**
	CD69%	-.6	**.03**
	CD90 MFI	-.54	**.057**
**PGE2**	IL-8	.86	**.001**
	IL-17	.57	**.03**
	IL-6	.52	**.06**
	IL-2	-.71	**.006**
**PGE2**	IFN-ɣ	-.6	**.032**
	CD25 MFI	.55	**.046**
	% of $CD4^+CD25^+$ T cells	-.57	**.03.**
**IL-2**	LAP	.73	**.012**
	IFN-ɣ	.6	**.033**
	IL-10	.57	**.034**
	CD26 MFI	.53	**.051**
**IL-8**	IL-17	.75	**.012**
	IL-6	.75	**.017**
	CD25 MFI	.75	**.015**
**IL-6**	IL-17	.68	**.009**
	CD25 MFI	.6	**.022**
	% of $CD4^+$ $CD69^+$ T cells	.6	**.026**

**IL-17**	% of $CD4^+$ $HLA\text{-}DR^+$ T cells	.62	**.018**
	HLA-DR MFI	.69	**.008**
**TGF-β1 (LAP)**	IFN-ɤ	.66	**.045**
	CD26 MFI	.77	**.007**
	CD4 MFI	.7	**.013**
	% of $CD4^+CD25^+$ T cells	.75	**.001**
	% of $CD4^+CD279^+$ T cells	.62	**.045**
**IFN-ɤ**	CD4 MFI	.55	**.055**
	% of $CD4^+CD25^+$ T cells	.58	**.04**
	CD26 MFI	.58	**.043**
**G0**	G1	.76	**.0002**
	G2	.69	**.002**
	IL-8	.87	**.001**
	IL-17	.77	**.001**
	PGE2	.68	**.008**
	IL-6	.66	**.009**
	CD69 MFI	.65	**.008**
	% of $CD4^+CD25^+$ T cells	-.72	**.001**
	CD26 MFI	-.6	**.014**
	HLA-DR MFI	-.67	**.005**
**G1**	TGF-β1 (LAP)	.77	**.007**
	IFN-ɤ	.57	**.043**

	IL-2	.55	**.043**
	CD26 MFI	.77	**.0005**
	CD4 MFI	.61	**.011**
	% of $CD4^{+}CD25^{+}$ T cells	.67	**.004**
	PGE2	-.62	**.02**
	CD69 MFI	-.53	**.039**
**G2**	TGF-β1 (LAP)	.89	**.002**
	IFN-ɤ	.6	**.035**
	IL-2	.53	**.055**
	CD26 MFI	.87	**<.0001**
	CD4 MFI	.78	**.0003**
	% of $CD4^{+}CD25^{+}$ T cells	.63	**.008**
	PGE2	-.6	**.025**

r: Spearman or Pearson correlation coefficient, BMI: body mass index, IL: interleukin, CC: coculture, ASCs: adipose stem/stromal cells, MFI: median fluorescence intensity, IDO, indoleamine 2,3 dioxygenase, IFN-ɤ: interferon gamma, CD: cluster of differentiation, PGE2: prostaglandin E2, TGF-β1: transforming growth factor beta 1.basal: expression in ASCs monoculture, G: division generation, %: percentage.

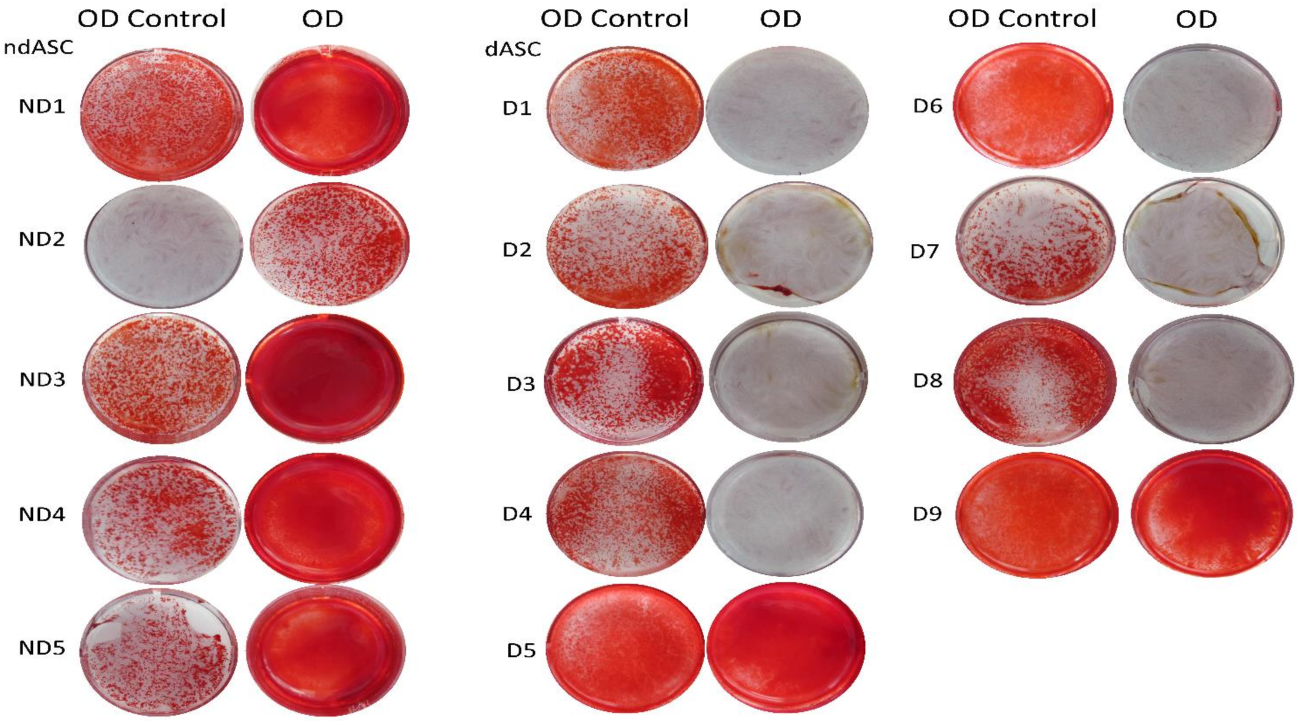

Supplementary Figure 1: Osteogenic differentiation of ASCs at passage 5 or 6. All ndASCs were able to differentiate toward osteogenic lineages, as shown by strong deposits of minerals and Alizarin Red staining when cultured in osteogenic conditions (OD). Only two out of nine dASCs (D5 and D9) were able to differentiate, and paradoxically, more mineral deposits were present in control conditions (OD control) than in OD for samples derived from donors D1-4 and D6-8. The images represent 24-well plate wells. ND: it abbreviates ASC line from nondiabetic donor. D: it abbreviates ASC line from donor with obesity and T2D. ndASCs: adipose stem/stromal cells from healthy donors. dASCs: adipose stem/stromal cells from donors with obesity and type 2 diabetes.

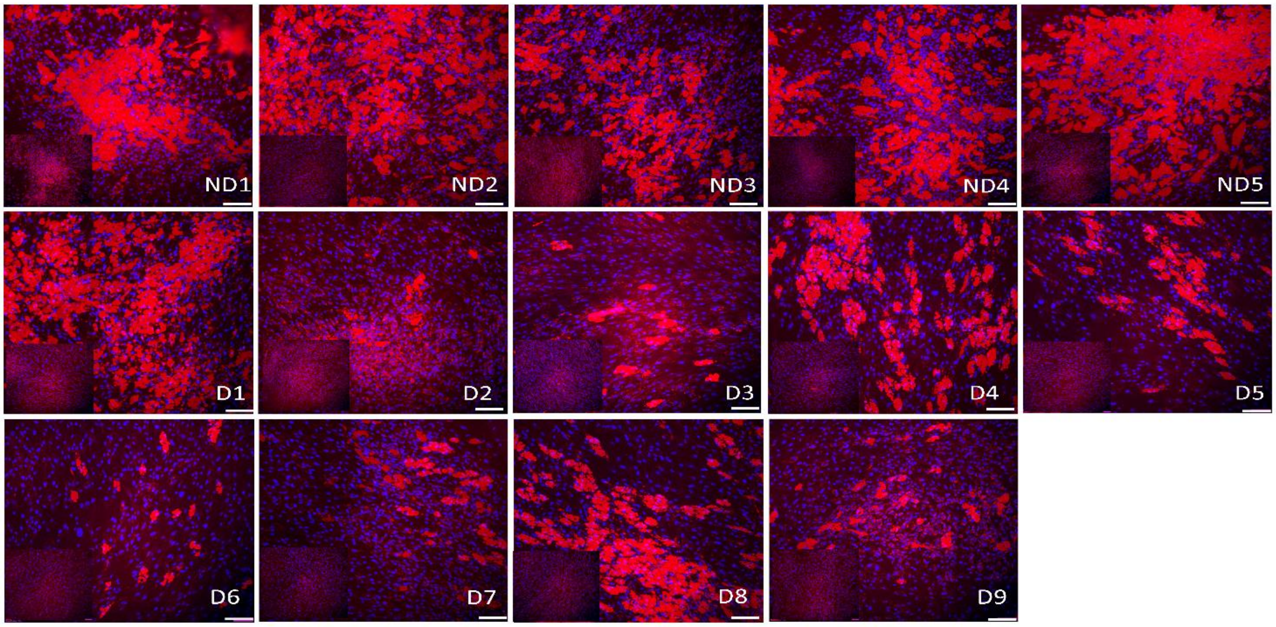

Supplementary Figure 2: Adipogenic differentiation (AD) of ASCs at passage 5 or 6. ndASCs and dASCs from all donors were able to differentiate toward the adipogenic lineage in the AD medium, as shown by the accumulated lipids and Oil Red O staining. Scale bar: 100 µm. ND: it abbreviates ASC line from nondiabetic donor. D: it abbreviates ASC line from donor with obesity and T2D. ndASCs: adipose stem/stromal cells from healthy donors. dASCs: adipose stem/stromal cells from donors with obesity and type 2 diabetes.

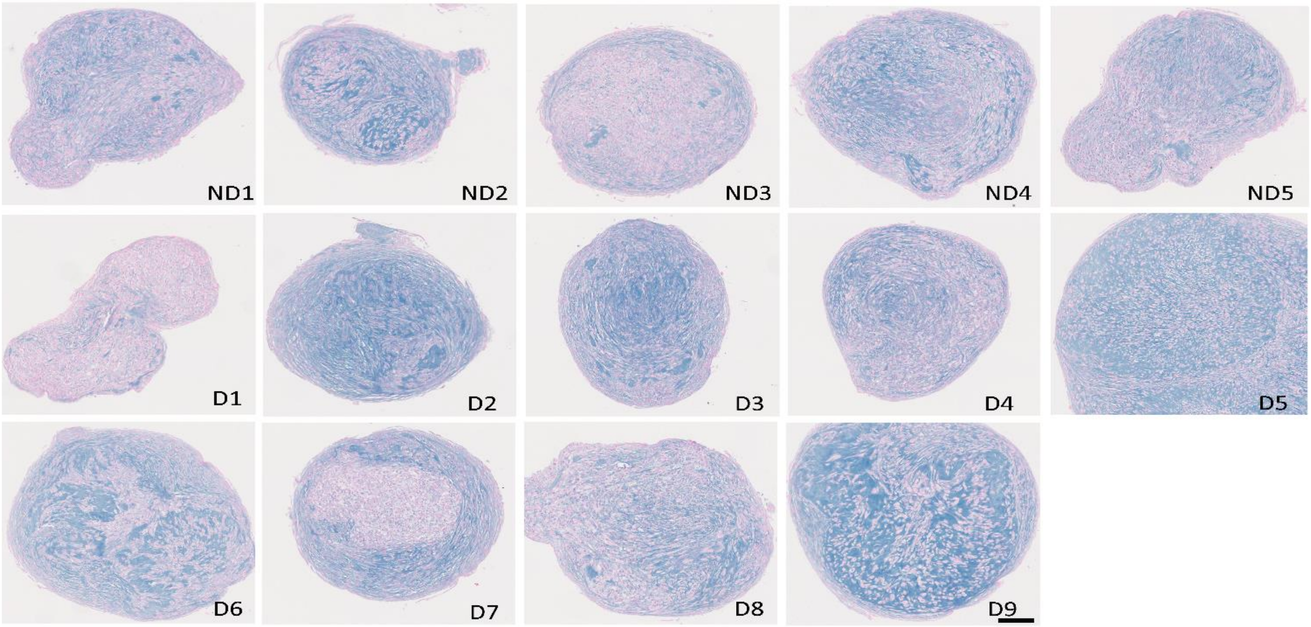

Supplementary Figure 3: Chondrogenic differentiation of ndASCs and dASCs at passage 5 or 6. The chondrogenic differentiation efficiency varied among the donors, and some ASC lines formed only small amounts of sulfated glycosaminoglycans (GAGs), as shown by Alcian Blue staining. The chondrogenic differentiation outcome was not consistently different between ndASCs and dASCs. Scale bar: 100 µm. ND: it abbreviates ASC line from nondiabetic donor. D: it abbreviates ASC line from donor with obesity and T2D. ndASCs: adipose stem/stromal cells from healthy donors. dASCs: adipose stem/stromal cells from donors with obesity and type 2 diabetes.

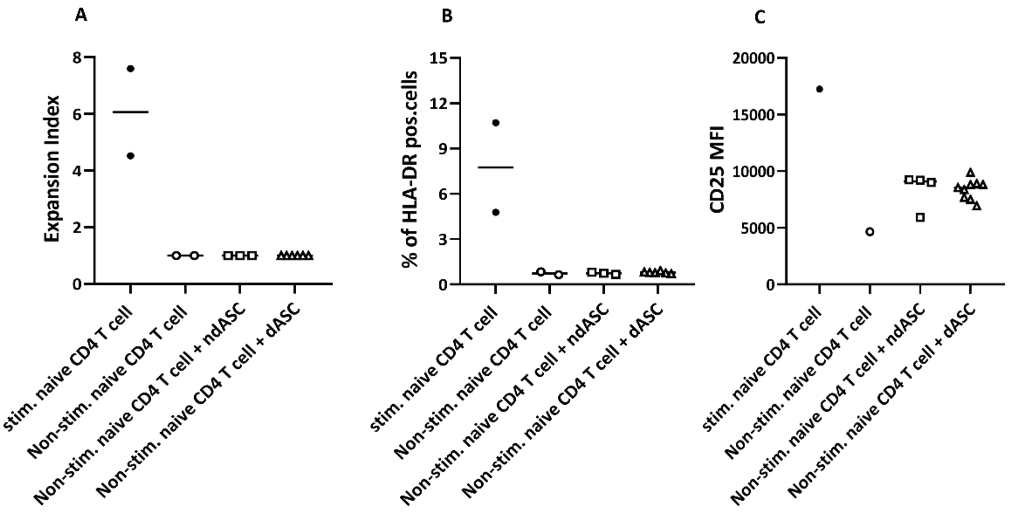

Supplementary Figure 4: Immunogenicity of ndASCs and dASCs. ndASCs (n=3) and dASCs (n=6) were cultured with a purified naïve CD4 T cell line (n=2) for six days at a ratio of 1:10 (ASCs:CD4^{+} T cells), and CTV-labeled CD4 T-cell proliferation and HLA-DR expression were then assessed by flow cytometry. The average of two CD4 T-cell line coculture experiments is indicated. Stimulated and nonstimulated naïve CD4 T-cell monocultures were used as positive and negative controls, respectively. ndASCs and dASCs did not induce proliferation (A) or HLA-DR expression (B) in naïve CD4 T cells. Increased CD25 expression (C) was detected in the ndASC and dASC cocultures compared to the nonstimulated monoculture. The horizontal lines represent the medians. Stim: stimulated. ndASCs: adipose stem/stromal cells from healthy donors. dASCs: adipose stem/stromal cells from donors with obesity and type 2 diabetes.

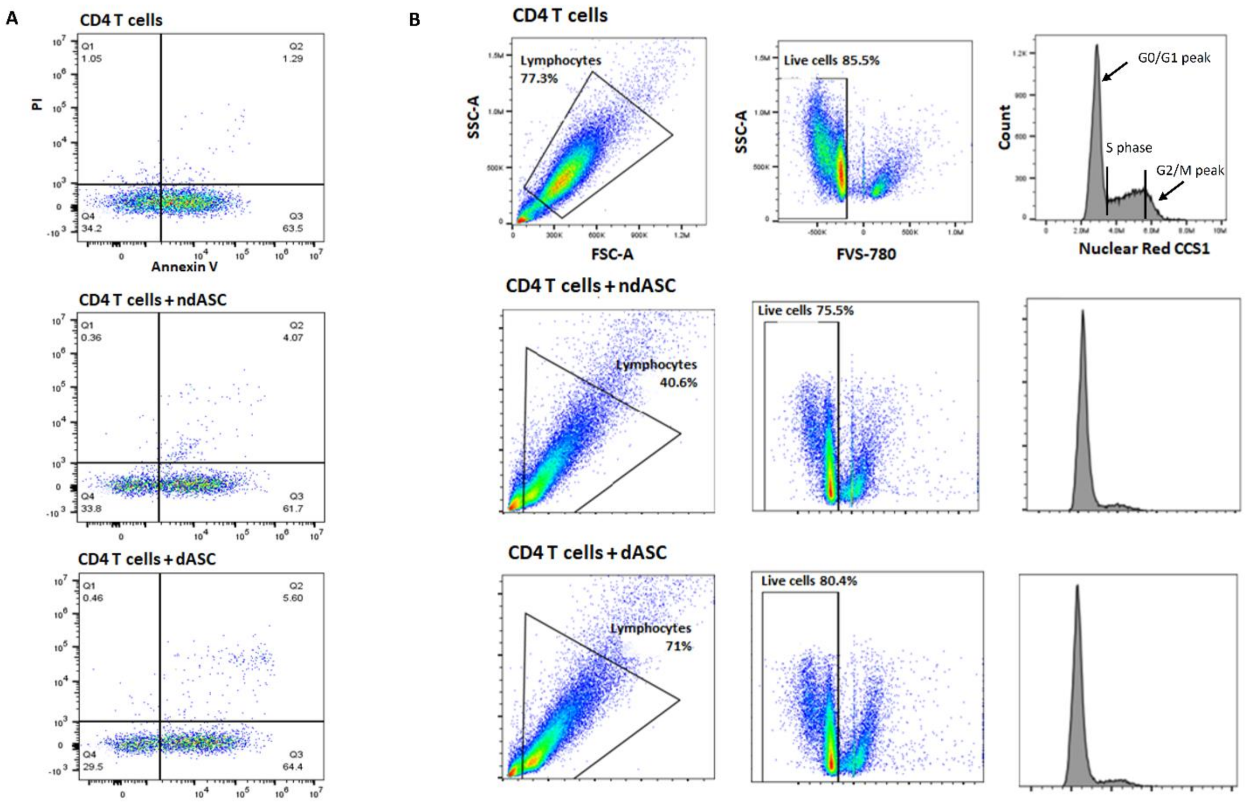

Supplementary Figure 5: (A) Representative flow cytometry plots for the significant upregulation of the late apoptotic CD4 T-cell proportion (Q2) by ndASCs or dASCs. (B) Representative flow cytometry plots showing the gating of viable CD4 T cells for the presentation of differential cell cycle phase distributions. ndASCs: adipose stem/stromal cells from healthy donors. dASCs: adipose stem/stromal cells from donors with obesity and type 2 diabetes.

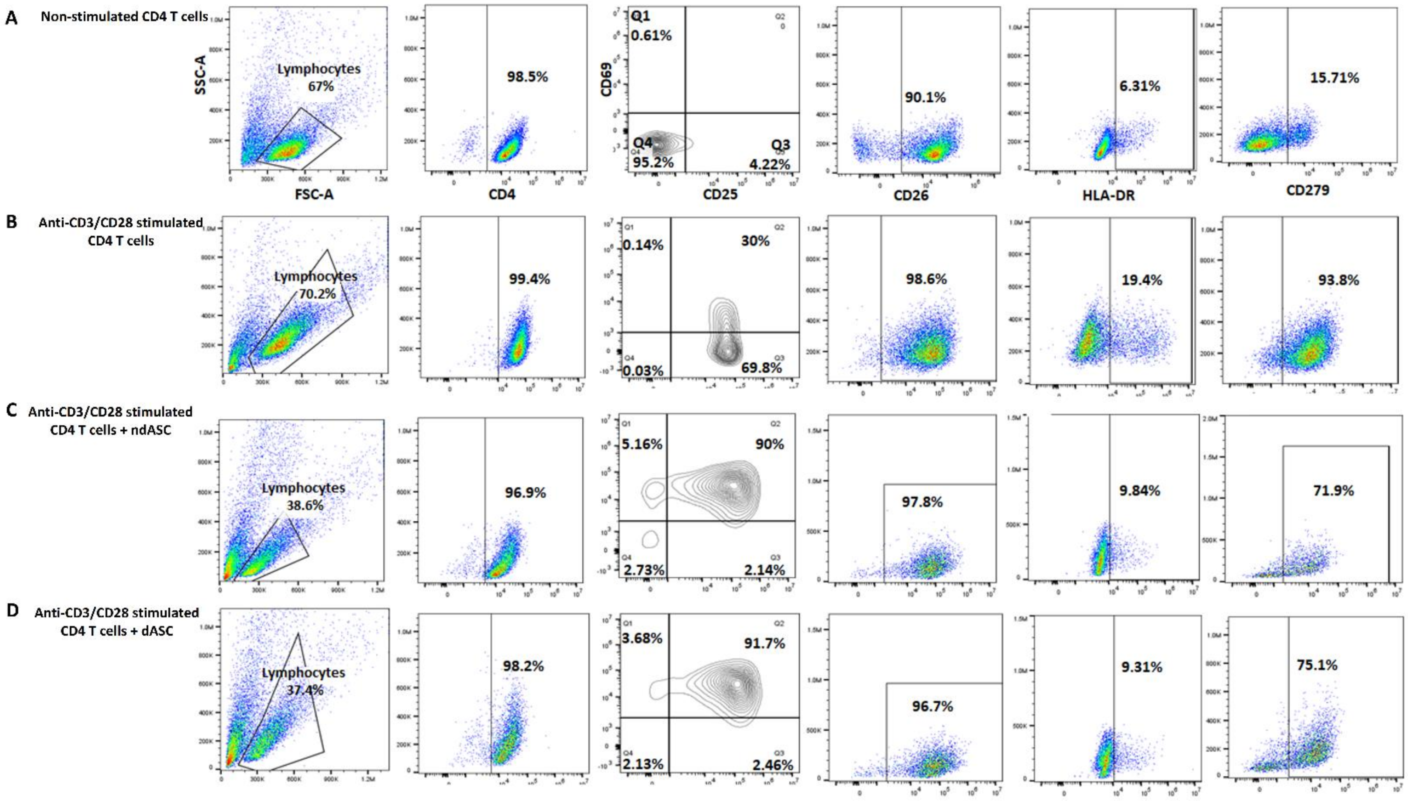

Supplementary Figure 6: Regulation of CD4 T-cell activation markers by ndASCs and dASCs. Representative flow cytometry plots for activation marker expression on CD4 T cells in control nonstimulated monocultures (A), control stimulated monocultures (B), or cocultures containing ndASCs (C) or dASCs (D). The plots show the changes in the physical properties of lymphocytes after activation and the noticeable increases in the positive-cell percentages for markers (CD69, CD25, CD26, HLA-DR, and CD279) (B vs. A). By coculture with ndASCs (C) or dASCs (D), the positive-cell percentages for CD26, HLA-DR, and CD279 were reduced; however, the proportion of the CD25 and CD69 double-positive population within the CD4 compartment (Q2 in the third column from left) was increased. ndASCs: adipose stem/stromal cells from healthy donors. dASCs: adipose stem/stromal cells from donors with obesity and type 2 diabetes.

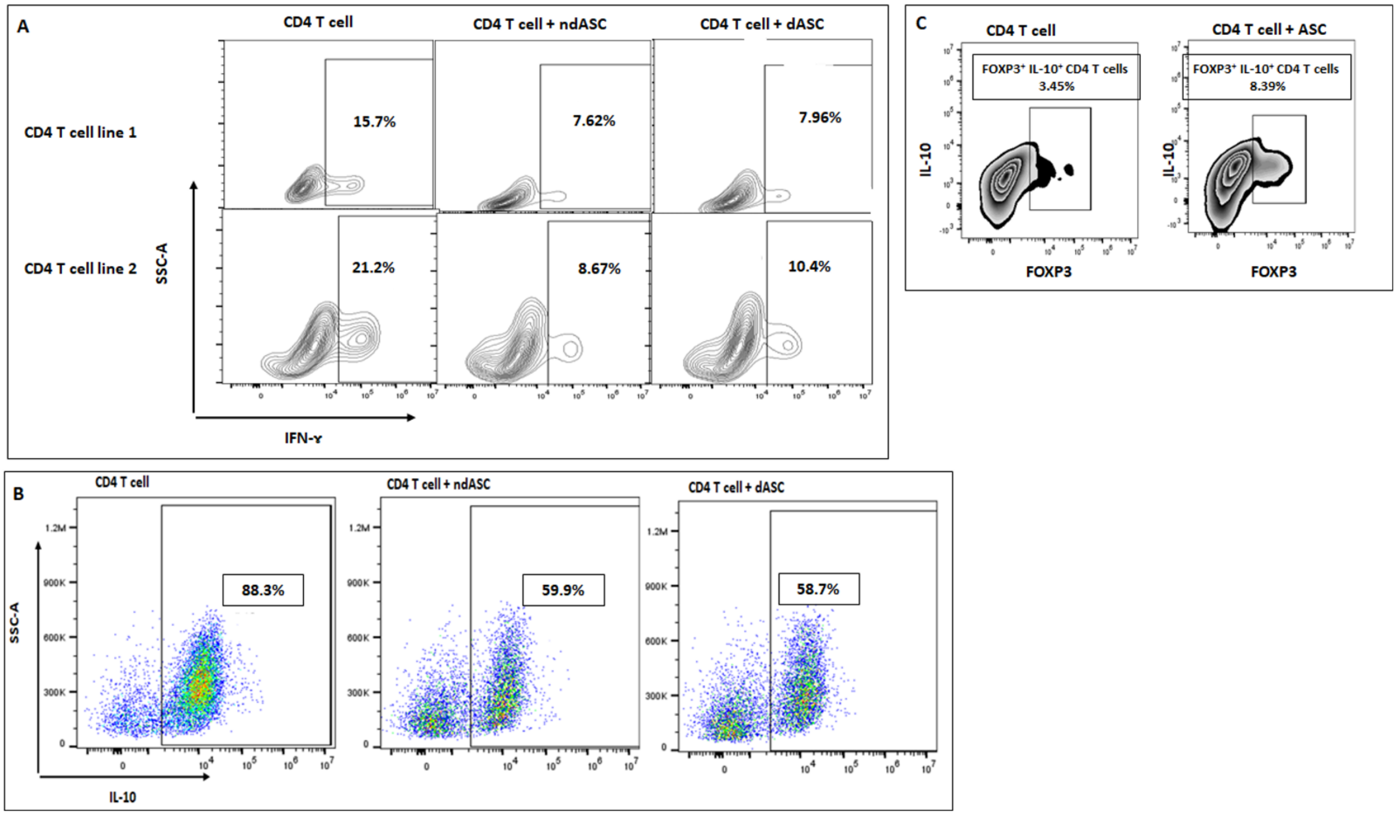

Supplementary Figure 7: (A) Representative flow cytometry plots showing the proportion of IFN-ɤ-positive cells within CD4 T cells in control monocultures and cocultures containing ndASCs or dASCs with two different T-cell lines. The overall IL-10-expressing cell percentage was reduced in the presence of ASCs (B); however, the potential of FOXP3$^+$ CD4$^+$ T cells to express IL-10 was enhanced by ASCs (C). ndASCs: adipose stem/stromal cells from healthy donors. dASCs: adipose stem/stromal cells from donors with obesity and type 2 diabetes.

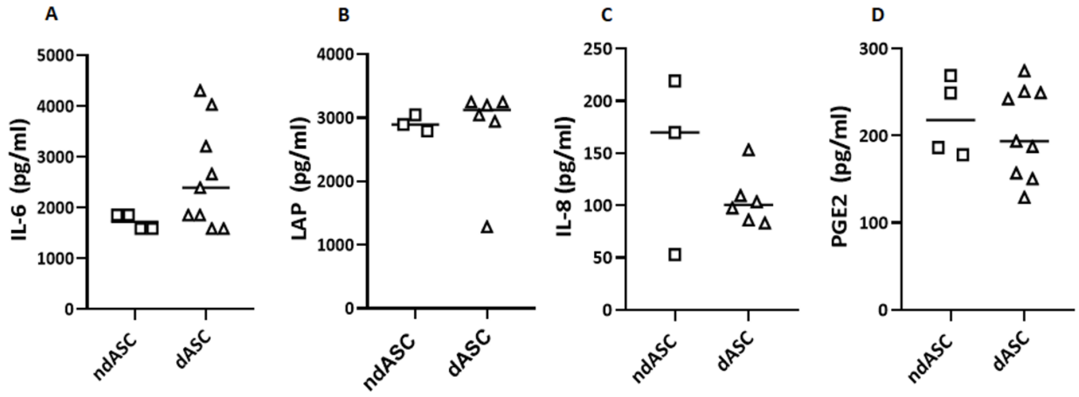

Supplementary Figure 8: Basal levels of IL-6, LAP, IL-8 and PEG2 secretion by ndASCs and dASCs. Considerable secretion of IL-6 (A) and LAP/TGF beta 1 (B) and lower levels of secretion of IL-8 (C) and PGE2 (D) were detected in the culture supernatants of ndASCs and dASCs cultured separately. The indicated cytokines were assessed in the supernatants of ndASC and dASC monocultures, which were established in parallel with the corresponding cocultures containing CD4 T cells. The Mann–Whitney test was used for statistical analysis. The horizontal lines represent the medians. ndASCs: adipose stem/stromal cells from healthy donors. dASCs: adipose stem/stromal cells from donors with obesity and type 2 diabetes.
